# Gestational diabetes is associated with change in the gut microbiota composition in third trimester of pregnancy and postpartum

**DOI:** 10.1186/s40168-018-0472-x

**Published:** 2018-05-15

**Authors:** Mie Korslund Wiinblad Crusell, Tue Haldor Hansen, Trine Nielsen, Kristine Højgaard Allin, Malte C. Rühlemann, Peter Damm, Henrik Vestergaard, Christina Rørbye, Niklas Rye Jørgensen, Ole Bjarne Christiansen, Femke-Anouska Heinsen, Andre Franke, Torben Hansen, Jeannet Lauenborg, Oluf Pedersen

**Affiliations:** 1 Novo Nordisk Foundation Center for Basic Metabolic Research, Section for Metabolic Genetics, Faculty of Health and Medical Science, University of Copenhagen, Blegdamsvej 3B, 2200 Copenhagen N, Denmark; 2 Department of Clinical Epidemiology, Bispebjerg and Frederiksberg Hospital, Hovedvejen 5, Nordre Fasanvej 57, 2000 Frederiksberg, Copenhagen Denmark; 3 Institute of Clinical Molecular Biology, Christian-Albrechts-University Kiel, University Hospital Schleswig Holstein, Campus Kiel, Rosalind-Franklin-Straße 12, 24105 Kiel, Germany; 4 Center for Pregnant Women with Diabetes, Department of Obstetrics, Rigshospitalet University Hospital, Blegdamsvej 9, 2100 Copenhagen Ø, Denmark; 5 Institute of Clinical Medicine, Faculty of Health and Medical Sciences, University of Copenhagen, Blegdamdsvej 3B, 2200 Copenhagen N, Denmark; 6 Steno Diabetes Center Copenhagen, Niels Steensens Vej 2, 2820 Gentofte, Denmark; 7 Department of Obstetrics and Gynaecology, Hvidovre University Hospital, Kettegaards Allé 30, 2650 Hvidovre, Denmark; 8 Department of Clinical Biochemistry, Rigshospitalet University Hospital, Blegdamsvej 9, 2100 Copenhagen Ø, Denmark; 9 OPEN, Odense Patient Data Explorative Network, Odense University Hospital/Institute of Clinical Research, University of Southern Denmark, J.B. Winsløws Vej 9 A, 3. sal, 5000 Odense, Denmark; 10 Department of Obstetrics and Gynaecology, Rigshospitalet University Hospital, Blegdamsvej 9, 2100 Copenhagen Ø, Denmark; 11 Fertility Clinic 4071, Rigshospitalet University Hospital, Blegdamsvej 9, 2100 Copenhagen Ø, Denmark; 12 Department of Obstetrics and Gynaecology, Aalborg University Hospital, Reberbansgade, 9000 Aalborg, Denmark; 13 Department of Obstetrics and Gynaecology, Herlev University Hospital, Herlev Ringvej 75, 2730 Herlev, Denmark

**Keywords:** Gut microbiota, Gestational diabetes, Pregnancy, Bacterial species, Body mass index, Glycaemic traits, Gestational hyperglycaemia

## Abstract

**Background:**

Imbalances of gut microbiota composition are linked to a range of metabolic perturbations. In the present study, we examined the gut microbiota of women with gestational diabetes mellitus (GDM) and normoglycaemic pregnant women in late pregnancy and about 8 months postpartum.

**Methods:**

Gut microbiota profiles of women with GDM (*n* = 50) and healthy (*n* = 157) pregnant women in the third trimester and 8 months postpartum were assessed by 16S rRNA gene amplicon sequencing of the V1-V2 region. Insulin and glucose homeostasis were evaluated by a 75 g 2-h oral glucose tolerance test during and after pregnancy.

**Results:**

Gut microbiota of women with GDM was aberrant at multiple levels, including phylum and genus levels, compared with normoglycaemic pregnant women. *Actinobacteria* at phylum level and *Collinsella*, *Rothia* and *Desulfovibrio* at genus level had a higher abundance in the GDM cohort. Difference in abundance of 17 species-level operational taxonomic units (OTUs) during pregnancy was associated with GDM. After adjustment for pre-pregnancy body mass index (BMI), 5 of the 17 OTUs showed differential abundance in the GDM cohort compared with the normoglycaemic pregnant women with enrichment of species annotated to *Faecalibacterium* and *Anaerotruncus* and depletion of species annotated to *Clostridium* (sensu stricto) and to *Veillonella*. OTUs assigned to *Akkermansia* were associated with lower insulin sensitivity while *Christensenella* OTUs were associated with higher fasting plasma glucose concentration. OTU richness and Shannon index decreased from late pregnancy to postpartum regardless of metabolic status. About 8 months after delivery, the microbiota of women with previous GDM was still characterised by an aberrant composition. Thirteen OTUs were differentially abundant in women with previous GDM compared with women with previous normoglycaemic pregnancy.

**Conclusion:**

GDM diagnosed in the third trimester of pregnancy is associated with a disrupted gut microbiota composition compared with normoglycaemic pregnant women, and 8 months after pregnancy, differences in the gut microbiota signatures are still detectable. The gut microbiota composition of women with GDM, both during and after pregnancy, resembles the aberrant microbiota composition reported in non-pregnant individuals with type 2 diabetes and associated intermediary metabolic traits.

**Electronic supplementary material:**

The online version of this article (10.1186/s40168-018-0472-x) contains supplementary material, which is available to authorized users.

## Background

Pregnancy induces metabolic and immunological changes, characterised by increased insulin resistance and immune tolerance against the foetus and placenta [1, 2]. In predisposed women, these physiological changes may lead to the development of gestational diabetes mellitus (GDM). GDM is defined as abnormal glucose regulation with onset or first recognition during pregnancy and is one of the most common complications during pregnancy with an incidence of 2–6% of all pregnancies in Europe [3, 4]. GDM is a transient state, but even though glucose regulation often normalises shortly after delivery, women with GDM have a 40% increased risk of developing type 2 diabetes mellitus within a 10–15-year period [3, 5].

The influence of the gut microbial community on human health is becoming increasingly evident, and it has been shown that patients with metabolic disorders such as obesity and type 2 diabetes differ in faecal microbiota composition from healthy individuals [6, 7]. Studies investigating the gut microbiota of healthy pregnant women have documented profound alterations in composition from the first to the third trimester [8, 9]. Koren et al. found an increased abundance of *Actinobacteria* and *Proteobacteria* and a decreased abundance of *Faecalibacterium* during the third trimester, resembling the composition reported in non-pregnant adults with metabolic syndrome. Interestingly, when germ-free mice were inoculated with faeces from healthy women who were pregnant in the third trimester, some metabolic changes were demonstrated. The mice exhibited increased weight gain, slightly lower insulin sensitivity and a more pronounced inflammatory response when compared with mice inoculated with faeces from healthy women who were pregnant in the first trimester [8]; a result partly similar to the effects observed in germ-free mice when inoculated with microbiota from non-pregnant obese human donors [10] and when adults with metabolic syndrome is inoculated with microbiota from lean non-pregnant donors [11]. In contrast to the pronounced changes during pregnancy, no substantial differences in the maternal microbial composition have been reported when comparing late pregnancy to the first months postpartum [8, 12].

Here, we hypothesise that GDM imposes changes in the faecal microbiota (here termed gut microbiota) during late pregnancy which are distinct from microbiota changes of normal pregnant women and that some microbiota aberrance remains postpartum in women with previous GDM. Hence, we aimed to investigate the differences in the gut microbiota and host metabolism between normoglycaemic pregnant women and women with newly diagnosed GDM in late pregnancy and on an average 8 months postpartum where insulin sensitivity and immune functions under normal circumstances have returned to a habitual stage.

## Methods

### Study population and design

During January 2014 to February 2015, we phenotyped at Herlev University Hospital in Denmark pregnant women who were referred to a 2-h 75-g oral glucose tolerance test (OGTT) in their third trimester (27–33 gestational weeks). Selected references to the hospitals were due to the presence of risk factors for GDM (family history of type 2 diabetes mellitus; previous delivery of a child weighing ≥ 4500 g at birth; glucosuria; pre-pregnancy BMI ≥ 27 kg/m^2^; known polycystic ovarian syndrome). In total, 790 women were invited to participate in the project of whom 213 women were included in the study as they fulfilled the inclusion criteria of being singleton pregnant (verified by an ultrasound scan), of Danish white origin and without diagnosed pre-eclampsia at the time of inclusion. Multipara women with a previous normoglycaemic pregnancy were included. Women were excluded if they had taken antibiotics within a period of 2 months before the first visit. All included participants were re-invited for a follow-up visit on an average of 8.8 months postpartum (Additional file 1: Figure S1).

### Anthropometrics and derived traits

Height was measured to the nearest 0.5 cm without shoes using a wall-mounted stadiometer (first visit only). Weight was measured to the nearest 0.1 kg on an electronic scale (TANITA BC-420MA, Tanita Corporation of America, IL, USA) without shoes and dressed in light indoor clothing. Using a non-expandable measuring tape, waist and hip circumferences were measured to the nearest 1 cm in an erect position midway between the iliac crest and the lower costal margin and at the level of the pubic symphysis, respectively. Body composition was assessed (postpartum only) using bioelectric impedance analysis (TANITA BC-420MA, USA). Pre-pregnancy weight was reported by the participants according to the weight noted in their pregnancy health records. Twenty-four women did not recall their weight before pregnancy or did not bring their pregnancy health record at the first visit. Body mass index (BMI) was calculated by dividing the weight in kilograms by the square of height in meters. Normal weight was defined as BMI < 25 kg/m^2^, overweight as BMI ≥ 25 kg/m^2^ and < 30 kg/m^2^ and obesity as BMI ≥ 30 kg/m^2^ [13]. Weight gain during pregnancy was calculated as the difference in measured weight obtained at the first visit and self-reported pre-pregnancy weight.

Blood pressure was recorded by an automatic sphygmomanometer (A&D Medical, Japan) as the mean of duplicate measurements in a sitting position after a 10-min rest. Gestational hypertension was defined as development of either a systolic blood pressure ≥ 140 mmHg, a diastolic blood pressure ≥ 90 mmHg or both after 20 weeks of gestation [14].

### Questionnaires, medical records and derived traits

Participants completed a questionnaire on health and lifestyle, including information on smoking and alcohol consumption, stool consistency on the Bristol stool scale and bowel habits and social and educational attainment. Physical activity was recorded using a validated instrument by questionnaire [15]. At both visits, dietary habits were recorded using a validated food frequency questionnaire (FFQ) [16]. Intensity-weighted activity levels were calculated as metabolic equivalent of task (MET) hours per week [17]. Participants were asked to bring their medication and dietary supplements at each visit, and exact daily dosages were recorded. Information about gestational age at previous birth, delivery method and antepartum, intrapartum or postpartum antibiotic treatment was obtained from the hospital birth records.

### Biochemistry and derived traits

At both visits, the women were examined in the morning following a 10-h overnight fast.

Venous blood was collected in the fasting state for hormonal and metabolic biomarkers. A standard 2-h OGTT with 75 g of glucose dissolved in 200 ml of water and sampling of venous blood at 0, 30, 60 and 120 min was conducted.

Plasma glucose was analysed by the glucose oxidase method using a colorimetric slide test on a Vitros 5600 system (Ortho Clinical Diagnostics, Raritan, NJ USA; coefficient of variation (CV) 6.1%) at the Department of Clinical Biochemistry Herlev University Hospital during pregnancy and at the Department of Clinical Biochemistry Rigshospitalet, Glostrup postpartum. Plasma insulin was measured on a Roche cobas e411 system using an enzyme-linked chemiluminescent immunoassay (Roche Diagnostics GmbH, Mannheim, Germany; CV 2.8%). High-sensitivity C-reactive protein (hsCRP) was measured on a Roche cobas c701 system using a particle-enhanced turbidimetric immunoassay (Roche Diagnostics GmbH, Mannheim, Germany) with an intra-assay CV of 0.7–2.3%.

Homeostasis model assessment of insulin resistance (HOMA-IR), insulinogenic index and disposition index were calculated according to Matsuda et al. (mmatsuda.diabetes-smc.jp/MIndex.html).

GDM was diagnosed according to the International Association of the Diabetes and Pregnancy Study Group (IADPSG) criteria: one or more elevated values—fasting plasma glucose ≥ 5.1 mmol/L and/or 1-h plasma glucose ≥ 10.0 mmol/L and/or 2-h plasma glucose ≥ 8.5 mmol/L [18].

### Descriptive analyses

Statistical analyses were performed using R Studio version 1.0.136 (http://www.r-project.org/). Two-tailed Student’s *t* test and the *χ*^2^ test were used to test the differences in phenotypical characteristics. Variables were log transformed to improve normality and homoscedasticity where appropriate. A Wilcoxon signed-rank test was applied for paired non-normally distributed data. Linear mixed models were used to compare dietary intake during pregnancy and postpartum.

### Microbiome analyses

#### DNA extraction, library preparation, sequencing and initial preparation of data

Faecal samples were collected at home by the participants within 48 h of the OGTT, following a standardised procedure including antiseptic handling, collection in sterile tubes and immediate freezing at − 18 °C. The samples were transferred to the laboratory on dry ice within 48 h of collection and stored at − 80 °C until DNA extraction. Two hundred nine women provided faecal samples at baseline, 122 of whom also provided a faecal sample postpartum.

We used the NucleoSpin Soil kit (Macherey-Nagel) to extract the total faecal genomic DNA from 150 to 200 mg of faecal material [19]. The faecal material was suspended in SL2 buffer containing SX enhancer, and cell disruption was carried out by bead beating at 30 Hz for 5 min using a TissueLyser instrument (Qiagen). Sufficient amounts of DNA could not be extracted from four faecal samples due to too little material.

Variable regions V1-V2 of the 16S rRNA gene were amplified using the 27F/338R primer pair [20]. The normalisation of PCR product was performed using the SequalPrep Normalization Plate Kit (Life Technologies). Pooling and sequencing were done on the Illumina MiSeq platform using a dual-indexing strategy [21] with MiSeq Reagent Kits v2. Demultiplexing was based on zero mismatches in the barcode sequence. The raw sequencing data was trimmed using sickle [22] in paired-end mode, using default quality values and a minimum read length of 100 bp after trimming. Forward and reverse read were merged using vsearch (v1.9, https://github.com/torognes/vsearch), setting minimal and maximal overlap to 280 and 350 bp, respectively. Further quality control included filtering based on a maximum expected error of 1 (vsearch v1.9) and a read quality below 30 in maximum 5% of the sequence, followed by reference-based chimera detection using vsearch. Quality controlled reads were annotated using UTAX [23], and sequences that could not be assigned to bacteria and sequences assigned to chloroplasts were removed. Picking of operational taxonomic units (OTUs) was performed using vsearch based on the non-rarefied dataset and an identity cut-off of 97%, excluding only singleton sequences prior to clustering. OTU picking was followed by a second step identifying chimeras, this time using a de novo approach. The reads of each sample were mapped to these OTU sequences, and 10,000 randomly chosen sequences were used to construct the OTU abundance table. Additionally, OTU sequences underwent taxonomic annotation using the ribosomal database project (RDP) classifier [24] and training set 16 provided on the RDP website (https://rdp.cme.msu.edu). The cut-off for annotation was set to a classification score of 0.8, assigning each OTU to the lowest taxonomic level exceeding this threshold in the classification. This classification was used to construct a taxonomic abundance table based on the OTU abundance table, collapsing OTUs assigned to the same taxon into one taxonomic bin. The final dataset of the third trimester pregnant women consisted of 209 samples (157 from normoglycaemic women, 50 from women with GDM and 2 from women with uncertain diabetes status due to missing information about fasting plasma glucose level) and 122 postpartum samples (79 and 43 from normoglycaemic and diabetic women, respectively).

#### Data analyses

Alpha diversity metrics (richness and Shannon’s index) were calculated using the *phyloseq* R package based on rarefied OTU counts. Pielou’s evenness index was calculated as Shannon’s index/ln (richness). The cross-sectional difference in alpha diversity between groups during pregnancy and postpartum was assessed using Student’s *t* test. Change in alpha diversity from pregnancy to postpartum in women with available samples from both time points was assessed using a mixed linear regression with a random effect of the subject, for only women with available samples at both time points. To test for differential change in alpha diversity metrics from pregnancy to postpartum in women with and without GDM, an ANOVA model was fitted with a two-way interaction between GDM status and time (factor with four levels) as the independent variable and the contrast Postpartum|Diabetic − Postpartum|Normoglycaemic − Pregnant|Diabetic + Pregnant|Normoglycaemic was tested using a post hoc *t* test. Models were fitted using restricted maximum likelihood, and assumptions were checked visually by inspection of residual plots (homoscedasticity) and normal probability plots (Gaussian distribution). Association between alpha diversity metrics and glycaemic traits (fasting and 2 h plasma glucose levels, insulin sensitivity and disposition index) was tested using linear regression with and without adjustment for pre-pregnancy BMI.

Analyses of community structure were performed using the *vegan* R packages. The cross-sectional difference in community structure between women with and without GDM and between women with GDM diagnosed by either fasting or stimulated hyperglycaemia or a combination of the two was assessed by permutational analysis of variance (PERMANOVA) of weighted UniFrac distances, as implemented in the *adonis* function. The association of glycaemic traits with community structure was also assessed using PERMANOVA of weighted UniFrac distances. Principal coordinate ordination was performed using the *capscale* function of the vegan package, specifying an unconstrained model. Vectors of glycaemic traits were fitted onto the ordination using the *envfit* function. For longitudinal analyses of community structure, PERMANOVA models were fitted with permutations constrained within each individual. Differential change in community structure from pregnancy to postpartum in women with and without GDM was assessed by contrasting the levels of the interaction between GDM status and time as described above.

To assess the cross-sectional differences at OTU level, we performed differential abundance analyses on unrarefied, untransformed OTU tables using a negative binomial Wald test as implemented in the DESeq2 R package [25]. Only OTUs present in at least 10% of samples and with a mean proportional abundance of 0.01% (561 of 5464 OTUs) were considered. OTUs exhibiting a differential change from pregnancy to postpartum in women with and without GDM were identified by a mixed linear model ANOVA, as described for alpha diversity metrics. For the longitudinal analyses, rarefied (10,000 sequences per sample) OTU abundances were added a pseudo count equal to the lowest non-zero abundance of each OTU and log transformed. Only the results significant at a 10% false discovery rate (*ad modum* Benjamini-Hochberg) are reported.

At deeper taxonomic levels (biomarkers from phylum to genus level), we performed linear discriminant analysis (LDA). Here, we applied LEfSe [26] with default parameters (alpha value for Wilcoxon tests was set at 0.05, the logarithmic LDA score threshold set at 2.0) to identify taxonomic biomarkers that characterise the differences between women with and without GDM and between pregnant women of different weight categories (normal weight, overweight and obese).

To test the association of individual OTUs with weight gain during pregnancy, a negative binomial Wald test as implemented in the *DESeq2* package was applied. Weight gain was adjusted for small variation in gestational age by linear regression and discretiszed by a tertile split. A negative binomial model was then specified with weight gain tertiles as the explanatory variable and pre-pregnancy BMI as a covariate (due to a strong inverse relationship between pre-pregnancy BMI and weight gain). Finally, the log_2_ fold difference in abundance between the upper and lower weight gain tertile was tested using a post hoc Wald test to circumvent the assumption of constant fold change for each unit change of the independent variable. The association of individual OTUs with glycaemic traits was tested using the same approach, contrasting the upper and lower tertiles of each trait, with and without adjustment for BMI.

The associations between deeper order taxa (phylum to genus level) and weight gain or glycaemic traits were tested using Spearman correlation of rarefied (10,000 reads) read counts with and without prior adjustment of the trait for pre-pregnancy BMI by linear regression. For weight gain, additional adjustment for gestational age by linear regression was performed prior to Spearman correlation analysis.

## Results

### Description of the study cohort

GDM was diagnosed in 50 of 213 women due to fasting (*n* = 25) or oral glucose-stimulated hyperglycaemia (*n* = 10) or a combination of the two (*n* = 15). As expected, markers of glucose and insulin homeostasis were higher in the GDM group compared with the group of normoglycaemic women (Table 1). Similarly, the Matsuda index of insulin sensitivity was lower, and the HOMA index of insulin resistance is higher in GDM (Table 1). Women with GDM had indications of diminished beta cell function as shown by lower insulinogenic index and disposition index. The women diagnosed with GDM had higher blood pressure (systolic 118 vs. 111 mmHg, *P* = 0.0004; diastolic 71 vs. 67 mmHg, *P* = 0.001) and pre-pregnancy BMI (29.3 vs. 27.1 kg/m^2^, *P* = 0.02) whereas the two groups were comparable in age and height (Table 1; Additional file 2: Table S1).

**Table 1:** Clinical variables of GDM and normoglycaemic women in the third trimester pregnancy

	GDM (*n* = 50)	Normoglycaemic (*n* = 161)	*P*
Descriptive measurements			
Age (years)	34.4 (4.4)	33.3 (4.6)	0.1
Gestational week at examination	28.7 (1.4)	28.4 (1.1)	0.1
Systolic BP (mmHg)	118 (11.2)	111 (10.6)	0.001
Diastolic BP (mmHg)	71 (8.1)	67 (8.5)	0.001
Height (cm)	168.6 (6.6)	169.7 (5.6)	0.2
Weight (kg)	91.8 (14.1)	87.3 (14.6)	0.06
Pre-pregnancy weight (kg) (*n* = 190)	83.5 (16.0) (*n* = 43)	78.48 (15.2) (*n* = 145)	0.06
Pre-pregnancy BMI kg/m^2^(*n* = 189)	29.3 (5.6) (*n* = 43)	27.1 (4.8) (*n* = 144)	0.02
Weight gain (kg)	7.4 (4.7)	8.8 (7.5)	0.3
Normal and excessive weight gain (*n*)	3 excessive (13.0%) 20 normal (87.0%)	17 excessive (16.2%) 88 normal (83.8%)	1
Biochemistry			
Glucose at time 0 min (mmol/L)	5.2 (0.4)	4.6 (0.2)	5.8 × 10^−31^
Glucose at time 30 min (mmol/L)	8.1 (1.0)	6.9 (0.9)	2.7 × 10^−12^
Glucose at time 60 min (mmol/L)	8.9 (1.3)	7.1 (1.2)	1.9 × 10^−15^
Glucose at time 120 min (mmol/L)	7.9 (1.4)	6.2 (1.0)	5.0 × 10^−17^
Insulin at time 0 min (pmol/L)	112.1 (40.6)	82.7 (39.6)	3.3 × 10^−5^
Insulin at time 30 min (pmol/L)	519.7 (1.6)	504.3 (1.7)	0.7
Insulin at time 60 min (pmol/L)	698.0 (1.8)	583.9 (1.7)	0.058
Insulin at time 120 min (pmol/L)	750.5 (1.8)	482.2 (1.7)	6.3 × 10^−6^
HbA1c (mmol/mol)	34.0 (3.1)	33.0 (2.8)	0.02
HOMA-IR	2.1 (0.8)	1.5 (0.7)	1.3 × 10^−6^
Matsuda index	3.5 (1.5)	5.3 (1.6)	3.0 × 10^−8^
Disposition index	4.1 (1.6)	7.8 (1.6)	9.2 × 10^−12^
Insulinogenic index	1.2 (1.6)	1.5 (1.6)	0.004
hsCRP (mg/L)	4.4 (1.9)	3.8 (2.2)	0.2

Data presented as mean (SD). For continuous variables, *P* was calculated by two-tailed *t* test, and for categorical variables, *P* was calculated by chi-square test or Fisher’s exact test

*BMI* body mass index, *BP* blood pressure, *GA* gestational age, *HbA1c* glycated haemoglobin, *HOMA-IR* homeostatic model of insulin resistance, *hsCRP* high sensitive C-reactive protein

More than 80% of women in either group took multivitamin supplements, and 2–3% took probiotic supplements (Additional file 2: Table S1). The two groups were comparable in terms of overall dietary intake based on the distribution of macronutrients, Bristol stool scale score and bowel movement frequency (Table 1; Additional file 2: Table S1; Additional file 1: Figure S2).

The primary indication for referral to OGTT was BMI ≥ 27 kg/m^2^ in both cohorts (50% in the GDM cohort and 46% in the normoglycaemic cohort). Overall, BMI ≥ 27 kg/m^2^ as the indication for OGTT accounted for 46.9%, family history of type 2 diabetes accounted for 20.9% and a combination of risk factors for 18.5%. Glucosuria, polycystic ovarian syndrome and previous child with birth weight ≥ 4500 g were indications in 1.9, 7.6 and 4.3% of the cases, respectively (Additional file 2: Table S2).

On an average of 8.8 months postpartum, 125 women were re-examined, 43 women with GDM and 82 women with normal glucose regulation during the preceding pregnancy (Table 2; Additional file 2: Table S3). Plasma glucose concentrations during OGTT were significantly higher in women with previous GDM. The group of women with previous GDM showed signs of insulin resistance featured by increased plasma insulin levels during the OGTT and decreased Matsuda index of insulin sensitivity and a relatively reduced insulin secretion by a decreased disposition index (Table 2). The two groups were comparable in markers of lipid metabolism, inflammation, dietary intake, breastfeeding, Bristol stool scale and bowel movements (Table 2; Additional file 2: Table S3; Additional file 1: Figure S3). About 33–35% of the women took oral anticonceptives or had an intrauterine hormonal contraception at the time of follow-up (Table 2). Overall, dietary intake was significantly higher during pregnancy compared with postpartum using mixed models analyses (*P* < 0.05 in all tests) (Additional file 2: Table S4). No differences were shown in age, BMI or parity between the women participating in the postpartum examinations compared with the women who dropped out of the study.

**Table 2:** Clinical variables of GDM and normoglycaemic women at postpartum approximately 8 months after delivery

	Previous GDM (*n* = 43)	Previous normoglycaemic (*n* = 82)	*P*
Descriptive measurements			
Systolic BP (mmHg)	120 (11.2)	116 (8.7)	0.04
Diastolic BP (mmHg)	79 (8.2)	75 (8.0)	0.03
BMI (kg/m^2^)	30.0 (5.9)	29.2 (5.0)	0.4
Weight (kg)	85.3 (16.9)	84.6 (15.4)	0.8
Fat (%)	41.3 (6.6)	40.2 (6.4)	0.3
Biochemistry			
Glucose at time 0 min (mmol/L)	5.4 (0.4) (*n* = 41)	5.1 (0.3) (*n* = 73)	3.7 × 10^−5^
Glucose at time 30 min (mmol/L)	7.7 (1.1)	7.0 (1.0)	0.001
Glucose at time 60 min (mmol/L)	7.7 (1.9)	6.6 (1.4)	0.004
Glucose at time 120 min (mmol/L)	6.5 (1.2)	6.0 (1.0)	0.025
Insulin at time 0 min (pmol/L)	63.7 (1.7)	54.1 (2.1)	0.1
Insulin at time 30 min (pmol/L)	376.8 (1.7)	307.1 (1.8)	0.05
Insulin at time 60 min (pmol/L)	433.9 (1.9)	303.8 (1.9)	0.004
Insulin at time 120 min (pmol/L)	298.0 (1.8)	235.9 (2.0)	0.05
HOMA2-IR	1.4 (0.7)	1.3 (0.9)	0.4
Matsuda Index	5.8 (1.6)	7.6 (1.8)	0.01
Disposition Index	6.6 (2.1)	8.9 (2.0)	0.05
Insulinogenic index	1.15 (1.9)	1.17 (1.9)	0.9

Data presented as mean (SD) and range. For continuous variables, *P* was calculated by two-tailed *t* test, and for categorical variables, *P* was calculated by chi-square test or Fisher’s exact test. Two women from the GDM group only had fasting blood samples taken at the follow-up visit. Eleven samples of fasting glucose were coagulated and rejected by the department investigating glucose

*BMI* body mass index, *BP* blood pressure, *GA* gestational age, *HOMA-IR* homeostatic model of insulin resistance

### Similar diversity and community structure in women with gestational diabetes and normal glucose regulation

At a rarefied sequencing depth of 10,000 reads, we did not find any difference in the number of observed OTUs, Shannon’s diversity index or Pielou’s evenness index between GDM and normoglycaemic women in the third trimester of pregnancy (Additional file 1: Figure S4). When regressing alpha diversity measures against glycaemic traits, we found a nominally significant association (*P* = 0.02) between the number of observed OTUs and beta cell function represented by the disposition index, but the association was insignificant at a 10% false discovery rate (FDR) (*Q* = 0.2) (Additional file 1: Figure S5).

**Fig. 1:**
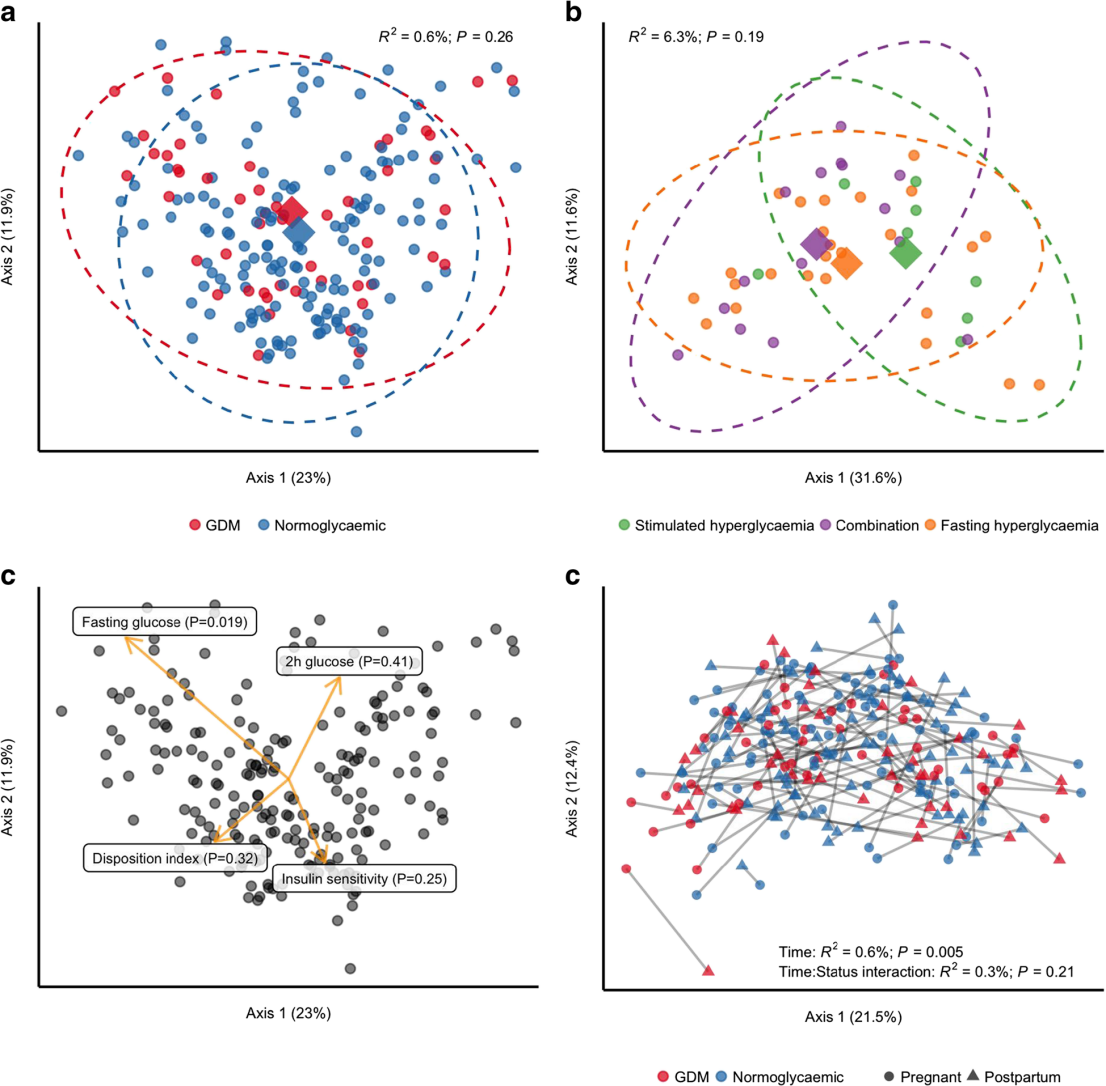
Community structure in women with and without gestational diabetes. For all analyses, samples were rarefied to an equal sequencing depth of 10,000 reads prior to principal coordinate (PCo) ordination based on weighted UniFrac distances. **a** Samples from pregnant women in the third trimester with (*n* = 50) or without (*n* = 157) gestational diabetes. Points are individual samples, and diamonds represent the average ordination scores and ellipses the 95% confidence intervals of a multivariate normal distribution of either group. *R*^2^ and *P* are from the permutational multivariate analysis of variance (PERMANOVA) as implemented in the *adonis* function of the vegan R package. **b** Community structure in pregnant women with gestational diabetes diagnosed by fasting hyperglycaemia (*n* = 25) or stimulated hyperglycaemia (2 h after an oral glucose challenge; *n* = 8), respectively, or by both (*n* = 15). Configuration is similar to panel **a**. **c** The association between glycaemic traits and community structure during pregnancy regardless of GDM status as determined by PERMANOVA. Vectors representing direction and magnitude of each trait were fitted onto the first and second PCo axes using the *envfit* function of the vegan R package. **d** Change in community structure from pregnancy to postpartum. Only samples from women examined at both time points are included (*n* = 43 and *n* = 79 for women with and without GDM, respectively). *R*^2^ and *P* are from PERMANOVA testing for a difference in community structure between samples collected during the third trimester and those collected postpartum and for a differential change in community structure in women with GDM compared to women without GDM

We also tested if GDM was associated with the overall community structure and found no difference in unweighted UniFrac distances (*R*^2^ = 0.6%; *P* = 0.26) between women with GDM and women with normal glucose regulation (Fig. 1a). As fasting hyperglycaemia and stimulated hyperglycaemia reflect two different pathophysiological processes [27], we also tested whether community structure differed in women with GDM diagnosed by either fasting or stimulated hyperglycaemia or a combination of the two, but we did not see any such association (Fig. 1b). However, when testing the association between fasting and stimulated glucose concentrations during pregnancy, we found a weak, nominally significant association between fasting plasma glucose levels and unweighted UniFrac distances (*R*^2^ = 1.0%; *P* = 0.037; Fig. 1c), but no association with stimulated 2-h plasma glucose level, insulin sensitivity or disposition index.

### Taxonomic biomarkers of gestational diabetes

Compositionally, the gut microbiota of the total group of pregnant women was dominated by members of the two major bacterial phyla *Firmicutes* and *Bacteroidetes*, which on average accounted for ~ 90% of all reads (Additional file 1: Figures S6 and S7). At the genus level, the composition followed a typical westernised pattern with *Bacteroides* as the predominant genus in women with gestational diabetes and normal glucose regulation alike (> 10% of all reads) and *Faecalibacterium*, *Prevotella* and unclassified *Lachnospiraceae* each accounting for > 5% of all reads on average, albeit as expected with substantial inter-individual variation (Additional file 1: Figure S8).

**Fig. 2:**
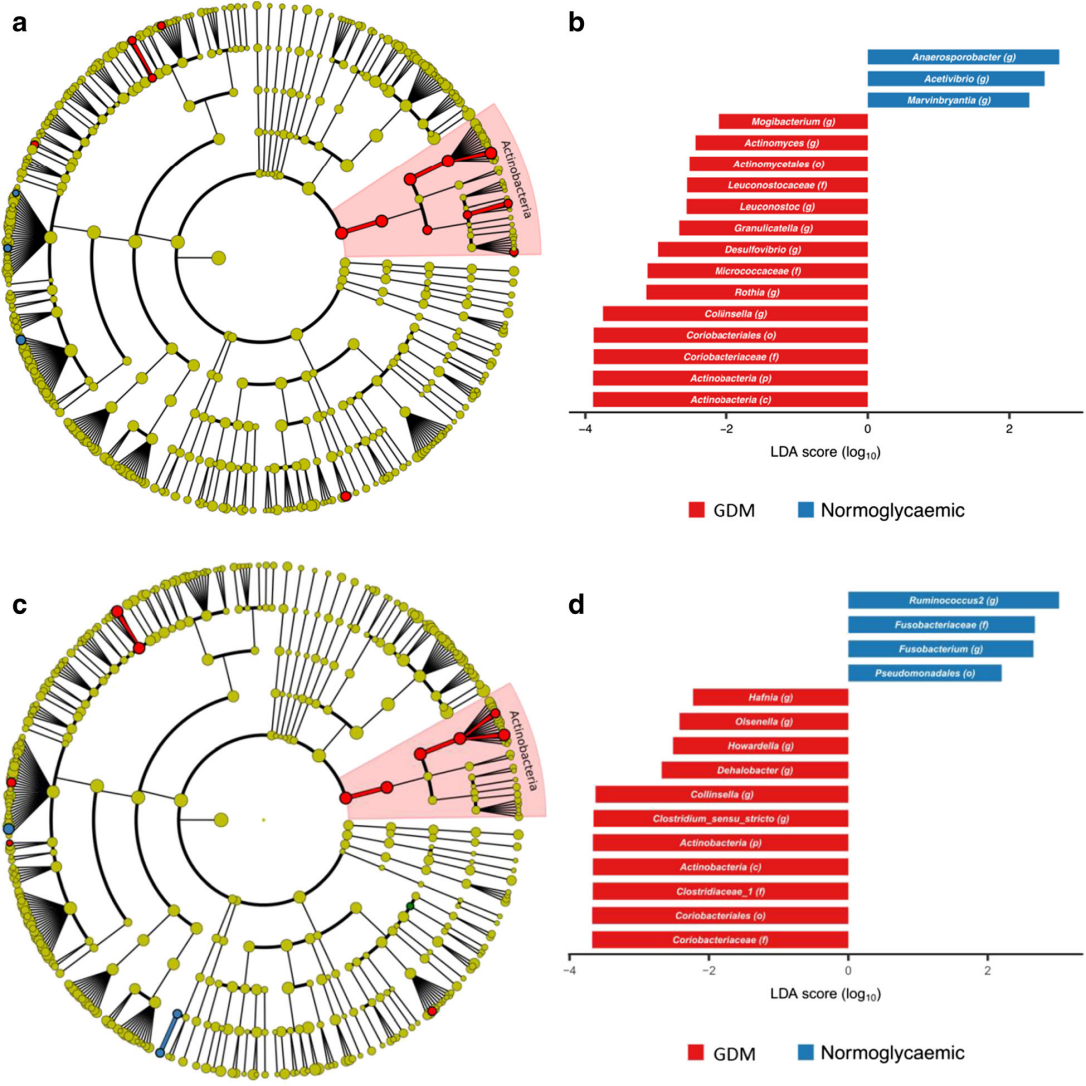
Taxonomic biomarkers of gestational diabetes during pregnancy and postpartum. Cladogram (**a**, **c**) and scores (**b**, **d**) of taxonomic biomarkers down to genus level identified by linear discriminant analysis (LDA) using LEfSe during pregnancy (**a**, **b**) and postpartum (**c**, **d**). Colour indicates the group in which a differentially abundant taxon is enriched (red: GDM and previous GDM; blue: normoglycaemic and previous normoglycaemic pregnancy)

Using linear discriminant analyses, we identified phylum *Actinobacteria* and several subordinate taxa as taxonomic biomarkers of GDM (Fig. 2a, b): genus *Collinsella* and the parent family *Coriobacteriaceae* and parent order *Coriobacteriales*; genus *Rothia* and the parent family *Micrococcaceae* and parent order *Actinomycetales*; and genus *Actinomyces*, also of order *Actinomycetales*. Within the phylum *Proteobacteria*, the genus *Desulfovibrio* was a biomarker of GDM. Within *Firmicutes*, the genus *Leuconostoc* and parent family *Leuconostocaceae*, genus *Granulicatella* and genus *Mogibacterium* were biomarkers of GDM, whereas the genera *Marvinbryantia*, *Acetivibrio* and *Anaerosporobacter* were markers of normal glucose regulation.

**Fig. 3:**
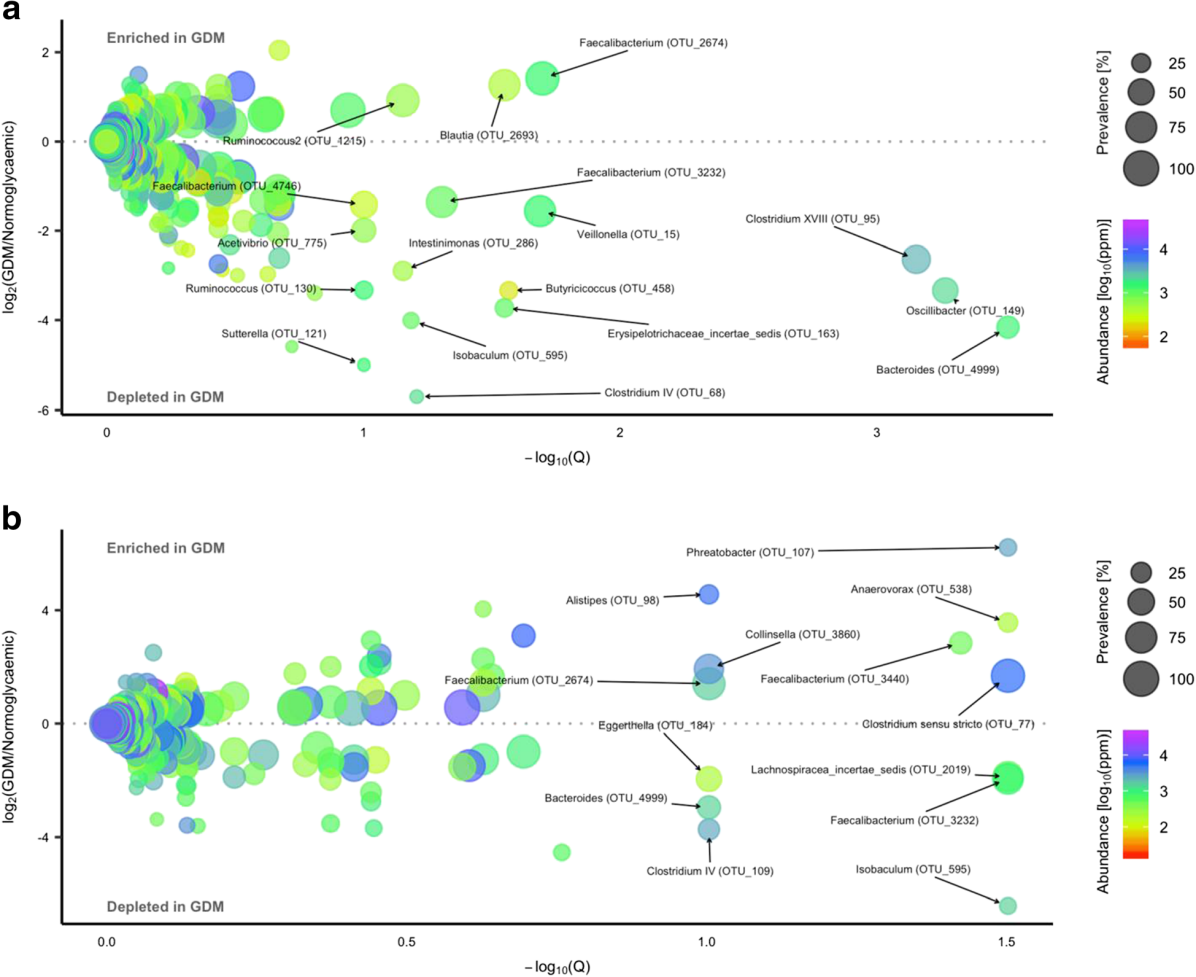
Operational taxonomic units differentially abundant during pregnancy and postpartum in women with GDM and normal glucose regulation. Volcano plot of estimated log_2_ fold difference in operational taxonomic unit (OTU) abundance between women with (*n* = 50) and without (*n* = 157) gestational diabetes during pregnancy (**a**) and postpartum (**b**) between women with (*n* = 43) and without (*n* = 79) previous gestational diabetes and corresponding Benjamini-Hochberg adjusted *P* values (*Q*) from negative binomial Wald tests as implemented in the DESeq2 R package. Prevalence indicates the percentage of participants in which a given OTU is present. Abundance indicates mean relative abundance (ppm) of a given OTU during the third trimester and postpartum. Names of OTUs differentially abundant at a 10% false discovery rate are given at the genus level

Using a negative binomial Wald test, we identified 17 species-level OTUs, predominantly within *Firmicutes* (15 out of 17), which in the third trimester were differentially abundant in women with GDM and women with normal glucose regulation at a false discovery rate of 10%. Three OTUs assigned to *Blautia* (OTU_2693), *Ruminococcus* (of family *Lachnospiraceae*; OTU_1215) and *Faecalibacterium* (OTU_ 2674) were enriched in women with gestational diabetes. In contrast, 14 OTUs assigned to *Acetivibrio* (OTU_775), *Intestinimonas* (OTU_286), *Erysipelotrichaceae incertae sedis* (OTU_163), *Isobaculum* (OTU_595), *Butyricicoccus* (OTU_458), *Clostridium IV* (*Ruminococcaceae*; OTU_68), *Clostridium XVIII* (*Erysipelotrichaceae*; OTU_95), *Oscillibacter* (OTU_149), *Ruminococcus* (*Ruminococcaceae*; OTU_130), *Bacteroides* (OTU_4999), *Veillonella* (OTU_15) and *Suterella* (OTU_121) and two assigned to *Faecalibacterium* (OTU_3232 and OTU_4746) were depleted in women with GDM (Fig. 3a; Additional file 2: Table S5).

### Association of microbial composition with glycaemic traits and inflammatory marker

**Fig. 4:**
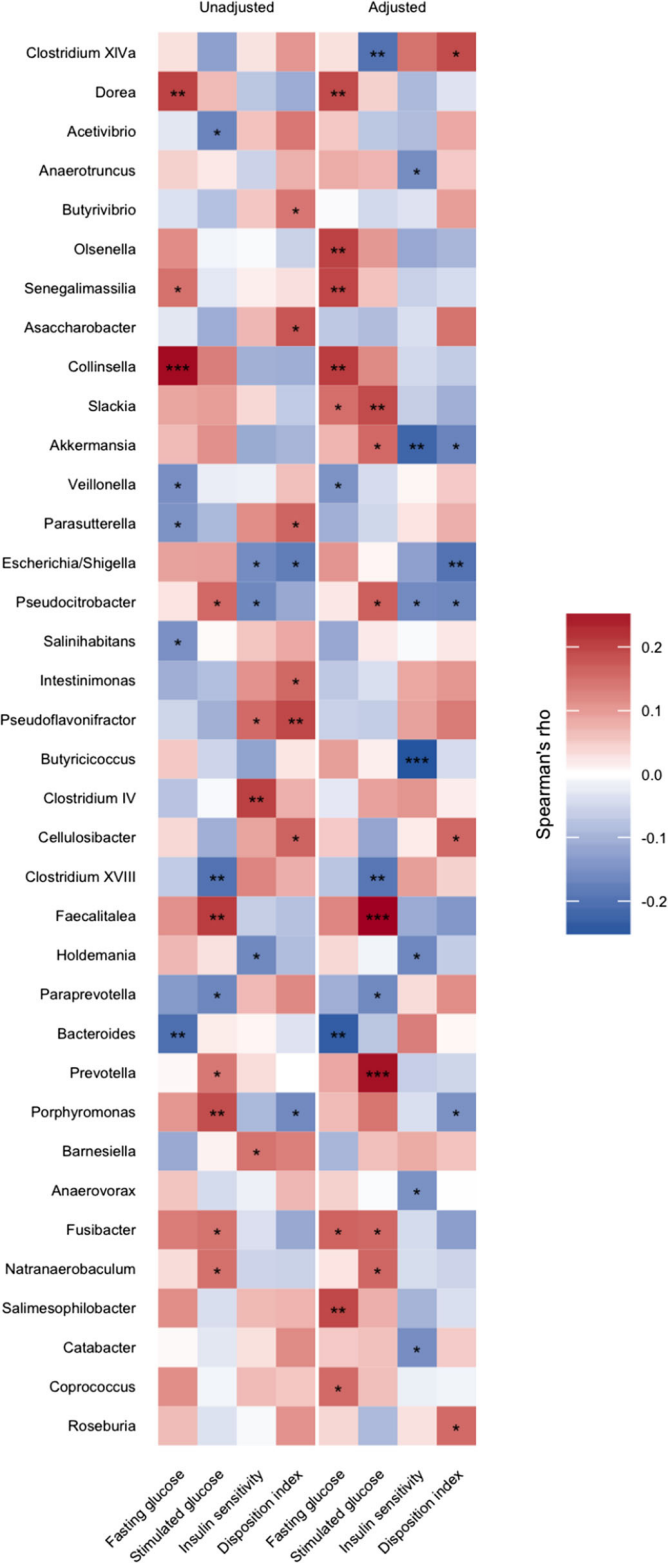
Bacterial genera associated with glycaemic traits during pregnancy regardless of GDM status. Heatmap of correlations (Spearman’s rho) between bacterial genera and fasting plasma glucose, stimulated 2-h glucose, insulin sensitivity and disposition index, with and without adjustment for body mass index. Only taxa nominally associated with either of the four traits are depicted. Taxa are ordered taxonomically. Results for all taxa are presented in Additional file 2: Table S5. *P ≤ 0.05, **P ≤ 0.01, ***P ≤ 0.001

We used Spearman correlation to identify deeper level taxa associated with glycaemic traits in pregnant women regardless of diabetes status and found that genus *Collinsella* and all parent taxa within *Actinobacteria* were positively correlated with fasting plasma glucose, but the association was abolished when adjusting for pre-pregnancy BMI (Fig. 4). BMI adjustment did, however, reveal a negative association between *Butyricicoccus* and insulin sensitivity (*r* = − 0.12; *Q* = 0.08) and positive correlations between stimulated 2-h plasma glucose level and *Prevotella* (*r* = 0.25; *Q* = 0.03) and *Faecalitalea* (*r* = 0.26; *Q* = 0.03). Also, associated with lower insulin sensitivity after adjustment for BMI were order *Verrucomicrobiales* and all parent taxa within *Verrucomicrobia*. *Akkermansia*, the dominant genus within *Verrucomicrobiales*, was nominally associated with lower insulin sensitivity (*r* = − 0.22; *P* = 0.003) adjusted for BMI, but the association was not significant at a 10% FDR (*Q* = 0.154).

In order to identify species-level OTUs associated with glycaemic traits, we divided the samples into tertiles according to the fasting plasma glucose level, stimulated 20-h plasma glucose level, insulin sensitivity index (Matsuda) and disposition index (as a measure of insulin sensitivity adjusted beta cell function) and contrasted the upper and lower tertiles of each trait. We identified 50 significant associations (FDR 10%) between 42 unique OTUs and the 4 glycaemic traits (Additional file 2: Table S6; Additional file 1: Figure S9). Nineteen were associated with stimulated 2-h plasma glucose level, 13 with insulin sensitivity and disposition index, respectively, and 5 were associated with a fasting plasma glucose level. Four OTUs assigned to the genus *Blautia* (OTU_2383, OTU_3654, OTU_140, OTU_2684) were associated with higher stimulated 2-h plasma glucose level. One of the four *Blautia* phylotypes (OTU_2383) was also associated with a higher fasting plasma glucose level and lower insulin sensitivity and disposition index, a pattern that was shared by the other three *Blautia* OTUs (OTU_3654, OTU_140, OTU_2684). One *Blautia* phylotype (OTU_486) exhibited the exact opposite pattern, being associated with lower fasting and stimulated 2-h plasma glucose levels and higher insulin sensitivity. Three *Escherichia*/*Shigella* phylotypes (OTU_680, OTU_361, OTU_3) were associated with higher disposition index and exhibited an identical pattern for the remaining traits (lower insulin sensitivity and higher fasting and stimulated 2-h plasma glucose levels).

Due to the strong association between pre-pregnancy obesity and microbial composition, we repeated the tests adjusting for pre-pregnancy BMI. Only ten associations between nine unique OTUs and the four glycaemic traits remained significant after the adjustment (Additional file 2: Table S6; Additional file 1: Figure S10). Notably, none of the 19 OTUs associated with stimulated 2-h plasma glucose level withstood the adjustment. Similarly, for insulin sensitivity, only 3 out of 13 associations remained; all three OTUs were associated with higher insulin sensitivity. Adjusting for pre-pregnancy BMI did, however, reveal several associations not previously detected, most of which involving *Clostridiales* species (12 of 17) and fasting plasma glucose level (10 of 17), including one *Christensenella* OTU (OTU_63) which was associated with higher fasting plasma glucose concentration. The strongest association, however, was between a *Clostridium IV* OTU (OTU_68) and lower 2-h plasma glucose level (log_2_ fold difference = − 25.45; *Q* = 1.1 × 10^−26^).

As the late pregnancy is defined as a pro-inflammatory state [1, 28], we wanted to investigate if differential taxa correlated with plasma levels of high-sensitive C-reactive protein (hsCRP) in pregnancy and postpartum, regardless of metabolic status. We used Spearman correlation to identify associations with higher order taxa; however, none of the findings withstood the adjustment for multiple testing (Additional file 2: Table S7). As with the maternal glycaemic traits, we divided the levels of plasma hsCRP into tertiles and contrasted the upper tertile to the lower in order to identify species-level OTUs that associated with plasma hsCRP during pregnancy and postpartum (Additional file 2: Table S8). Interestingly, *Bacteroides* (OTU_4999) both during pregnancy and postpartum is associated with higher levels of plasma hsCRP, whereas *Alistipes* (OTU_98) and *Anaerovorax* (OTU_538) are associated with lower levels of plasma hsCRP at both time points.

### Partial confounding effect of BMI

Overweight and obesity are known risk factors for GDM [29], and increased BMI has been associated with gut microbiota disruption in both pregnant [30, 31] and non-pregnant women [32]. In light of this and the overrepresentation of obesity among women with gestational diabetes (46.5 vs. 27.0% of women with normal glucose regulation (Additional file 1: Figure S11) in our cohort, we looked for microbial biomarkers of excess body weight in order to assess the potential confounding effect of BMI.

Comparing pregnant women with pre-pregnancy BMI within the overweight range (25–29.9 kg/m^2^) to women with normal (< 25 kg/m^2^) pre-pregnancy BMI, we identified *Parabacteroides* and *Porphyromonas* as biomarkers of overweight (Additional file 1: Figure S12), whereas *Akkermansia* and all parent taxa within *Verrucomicrobia* were markers of normal weight, as were the genera *Eggerthella* and *Sporobacter*, as well as genera *Ethanoligenens* and *Clostridium XVIII* (*Erysipelotrichaceae*).

Comparing pregnant women with pre-pregnancy obesity (BMI ≥ 30 kg/m^2^) to women with normal (< 25 kg/m^2^) pre-pregnancy BMI identified the genera *Porphyromonas*, *Acidaminococcus* and *Ruminococcus* (*Lachnospiraceae*) as markers of obesity and the genera *Ethanoligenens*, *Sporobacter*, and *Eggerthella*, as well as an unclassified *Erysipelotrichaceae* genus, as markers of normal weight. Notably, there was no overlap between the taxonomic biomarkers of GDM and the markers of overweight and obesity.

Disregarding the weight categories by correlating pre-pregnancy BMI with taxa abundances confirmed the results, showing that genus *Acetivibrio* (*r* = − 0.16; *P* = 0.03) and genus *Leuconostoc* (*r* = 0.15; *P* = 0.04) were the only GDM discriminant taxa nominally associated with pre-pregnancy BMI (Additional file 2: Table S9).

At OTU level, we primarily found that OTUs are differentially abundant comparing women with pre-pregnancy normal weight to women that were obese pre-pregnancy. Ten OTUs were enriched in the obese women, and 12 OTUs were depleted compared to pre-pregnancy lean women. Only three OTUs showed differential abundance between pre-pregnancy lean women and pre-pregnancy overweight women (Additional file 2: Table S10; Additional file 1: Figure S13).

At OTU level, adjusting for pre-pregnancy BMI reduced the number of differentially abundant OTUs to five, two of which were enriched in women with GDM and three of which were depleted (Additional file 2: Table S11; Additional file 1: Figure S14). Of those enriched in women with GDM in the full cohort, only one assigned to *Faecalibacterium* (OTU_2674) remained after the adjustment, whereas two depleted in women with GDM, both assigned to *Veillonella* (OTU_1626 and OTU_15), remained. One OTU assigned to *Anaerotruncus* (OTU_674) was enriched in women with GDM following adjustment for pre-pregnancy BMI, and another OTU assigned to *Clostridium* (sensu stricto) (OTU_1712) was depleted, neither of which were among the OTUs differentially abundant in the full cohort. They were, however, differentially abundant in the subcohort of women with known pre-pregnancy BMI (43 with GDM and 143 with normal glucose regulation) in which the adjusted analyses were conducted (Additional file 2: Table S11; Additional file 1: Figure S14A).

Adjusting for BMI did not affect the comparison of alpha diversity measures between women with GDM and with normal glucose regulation (*P* = 0.40, 0.81 and 0.86 for observed OTUs, Shannon’s diversity and Pielou’s evenness, respectively). Besides abolishing the nominal association between disposition index and observed OTUs, and revealing a nominally significant association between observed OTUs and post-load 2-h glucose level, adjusting for pre-pregnancy BMI had only a minor effect on the relationship between glycaemic traits and alpha diversity measures (Additional file 1: Figure S15).

### Third trimester gut microbiota associated with weight gain during pregnancy

Increased weight gain during pregnancy has been linked to an increased risk of developing GDM [33]. In our cohort, women gained on average 8.7 ± 3.8 kg (mean ± SD) leading up to the first visit during the third trimester, with no difference between women with GDM and women with normal glucose regulation (1.08 kg; 95%CI − 0.14–2.29) when adjusting for pre-pregnancy BMI and gestational age. Disregarding diabetes status, we identified 11 OTUs, mostly *Clostridiales* (7 of 11) species, associated with gestational weight gain. Seven OTUs were associated with lower weight gain, including a *Christensenella* OTU (OTU_63) and an *Alistipes* OTU (OTU_128). Four OTUs were associated with higher weight gain, including an *Eisenbergiella* OTU (OTU_258) and a *Lactobacillus* OTU (OTU_80) (Additional file 2: Table S12). Nine higher order taxa, predominantly *Firmicutes* (8 of 9), where associated with gestational weight gain at a nominal significance threshold. Among these, the genera *Coprococcus*, *Roseburia*, *Butyricicoccus* and *Clostridium* (sensu stricto) were positively correlated, and *Fusibacter*, *Holdemania* and an unclassified *Erysipelotrichaceae* genus were inversely correlated (Additional file 2: Table S13), yet none withstood correction for multiple testing.

### Changes in diversity, community structure and composition from pregnancy to postpartum

**Fig. 5:**
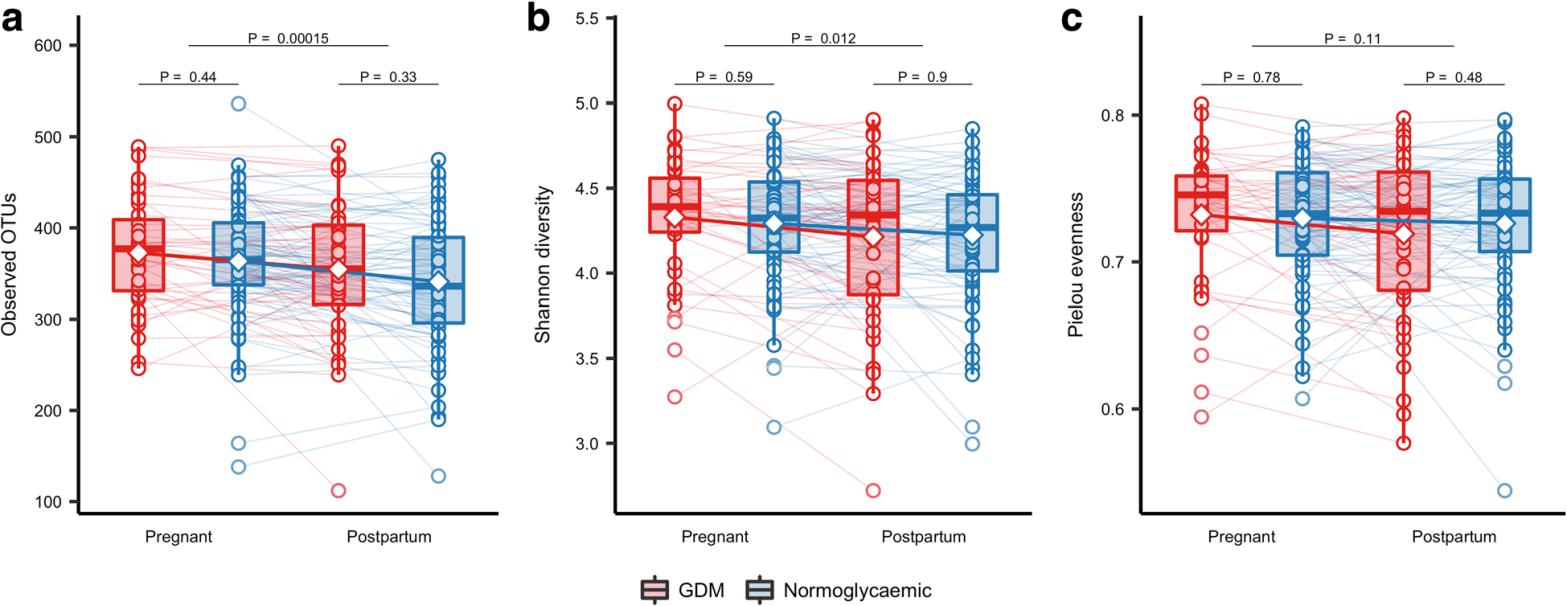
Change in alpha diversity from the third trimester to postpartum. Alpha diversity in pregnancy and postpartum as represented by observed richness (**a**), Shannon diversity (**b**) and Pielou evenness (**c**) based on the samples from GDM (*n* = 43) and normoglycaemic (*n* = 79) women with available faecal samples from the third trimester and 8 months postpartum. Samples were rarefied to an equal sequencing depth of 10,000 reads. Boxes represent interquartile range (IQR), with the inside line representing the median. Whiskers represent values within 1.5 × IQR of the first and third quartiles. Circles represent individual samples with lines connecting the samples from the same individual. Differences between GDM and normoglycaemic pregnancies within each time point were tested using Student’s *t* test. Difference in richness, Shannon diversity and Pielou evenness between time points in GDM and normoglycaemic women combined was tested using a mixed linear regression with a random effect of the subject in women with available samples at both time points

At an average of 8.8 months postpartum, 125 women were re-examined of which 43 women with previous GDM and 79 women with normal glucose regulation pregnancies volunteered stool samples. Similar to the third trimester, we did not see any difference in alpha diversity (observed OTUs, Shannon’s diversity and Pielou’s evenness) between women with preceding GDM and preceding normal pregnancy (Fig. 5). However, when studying the changes from pregnancy to postpartum using linear mixed regression modelling and accounting for the repeated nature of measurements performed on the same individual over time, we found a statistically significant reduction in the number of observed OTUs (*P* = 0.0002) and Shannon’s diversity (*P* = 0.012) irrespective of diabetes status (Fig. 5). Still, there was no change in Pielou’s evenness index (*P* = 0.11). Similarly, for beta diversity, we found a small significant change in community structure as represented by weighted UniFrac distances (*R*^2^ = 0.6%; *P* = 0.005) and unweighted UniFrac distances (*R*^2^ = 0.5%; *P* = 0.001), irrespective of diabetes status. Principal coordinate ordination showed that the variation over time did not reflect a uniform structural shift, but individual changes in community structure (Fig. 1d), changes which were independent of diabetes status.

Applying linear discriminant analyses, we identified the genera *Collinsella* and *Olsenella* and all parent taxa within phylum *Actinobacteria* as biomarkers of previous GDM. Genus *Clostridium* sensu stricto and the parent family *Clostridiaceae_1*, as well as the genera *Hafnia*, *Howardella* and *Dehalobacter*, were also biomarkers of previous GDM, whereas *Fusobacterium* and the parent family *Fusobacteriaceae* and genus *Ruminococcus2* (*Ruminococcus* of family *Lachospiraceae*) were markers of a previous normoglycaemic pregnancy (Fig. 2c, d).

Postpartum, we identified 13 OTUs, which were differentially abundant in women with preceding GDM and women with a normal glucose regulation pregnancy. Seven OTUs were enriched in women with previous GDM, including two *Faecalibacterium* OTUs, one of which (OTU_2674) was also enriched in women with GDM during pregnancy. Six OTUs were depleted postpartum in women with preceding GDM compared with women with a previous normal glucose regulation pregnancy, including a *Faecalibacterium* (OTU_3232), a *Bacteroides* (OTU_4999), and an *Isobaculum* OTU (OTU_595), which were also depleted in women with GDM during pregnancy (Fig. 3b).

**Fig. 6:**
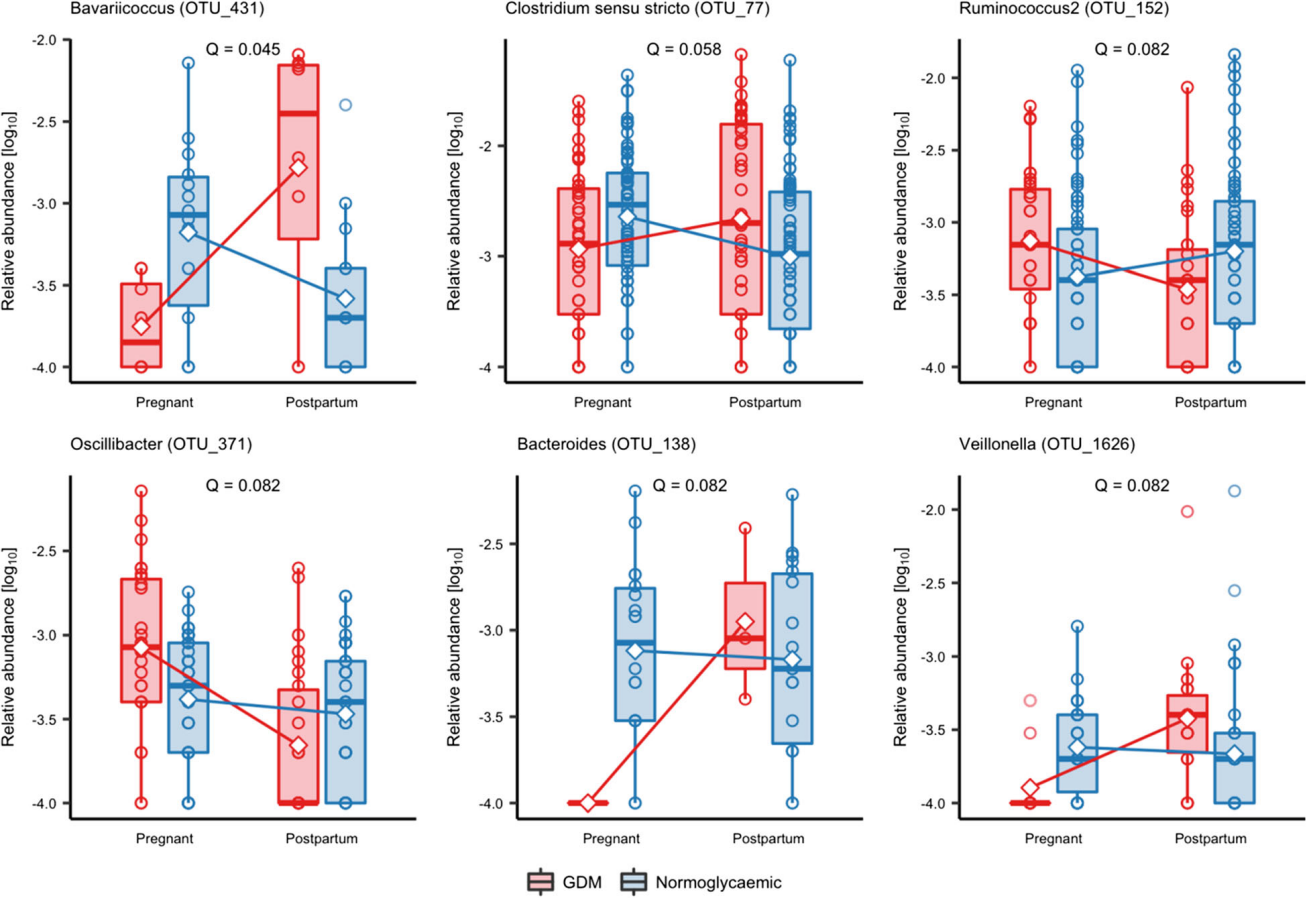
Operational taxonomic units exhibiting differential change from antepartum to postpartum in gestational diabetes and normoglycaemic women. Based on the samples from GDM (*n* = 43) and normoglycaemic (*n* = 79) women with available faecal samples from the third trimester and 8 months postpartum. Samples were rarefied to an equal sequencing depth of 10,000 reads. Boxes represent interquartile range (IQR), with the inside line representing the median. Whiskers represent values within 1.5 × IQR of the first and third quartiles. Circles represent individual samples. Change from the third trimester to 8 months postpartum between GDM and normoglycaemic women was modelled using mixed linear regression ANOVA of the interaction between GDM status and time, and a post hoc *t* test was used to test the difference in change between GDM and normoglycaemic women. Only the results significant at a false discovery rate of 10% are depicted. A full list of all nominally significant results is available in Additional file 2: Table S5

Using linear mixed regression model, we identified six OTUs which exhibited a differential change from antepartum to postpartum in women with preceding GDM and normal pregnancy at a 10% FDR. Compared with abundances during pregnancy, four OTUs assigned to *Bavariicoccus* (OTU_431), *Clostridium* sensu stricto (OTU_77), *Bacteroides* (OTU_138) and *Veillonella* (OTU_1626), respectively, were enriched postpartum in women with gestational diabetes but less abundant postpartum in women with normal glucose regulation. Contrastingly, two OTUs assigned to *Ruminococcus* (OTU_152) (*Lachnospiraceae*) and *Oscillibacter* (OTU_371) were depleted postpartum in women with GDM but enriched in women with a preceding normal pregnancy (Fig. 6; Additional file 2: Table S14). Seven higher order taxa, including the genera *Ruminococcus* (*Lachnospiraceae*), *Romboutsia*, *Clostridium* (sensu stricto), *Alloprevotella*, *Veillonella*, *Hydrogenoanaerobacterium* and *Coprobacillus*, exhibited differential change at a nominal significance threshold, but none withstood the correction for multiple testing (Additional file 2: Table S15).

## Discussion

Our study of women with GDM and normoglycaemic pregnant women demonstrated that GDM diagnosed in late pregnancy is associated with an aberrant gut microbial composition at the time of diagnosis. About 8 months postpartum, the gut microbiota of previous GDM women is still different from women who had a normal pregnancy.

Interestingly, we found that OTU richness was higher during late pregnancy compared with postpartum. Late normal pregnancy is characterised by increased insulin resistance and increased circulating levels of pro-inflammatory cytokines [1]. A reduced richness of intestinal microbiota has previously been associated with elevated pro-inflammatory markers and insulin resistance [34]. Our findings of higher alpha diversity in late pregnancy compared with postpartum could be influenced by the difference in diet during pregnancy compared with postpartum, as changes in nutrient composition and caloric intake are known to affect gut microbiota in humans [35, 36]. Expectedly, total energy intake was lower in postpartum regardless of GDM status, but we did not observe any major difference in the composition of relative macronutrients intake between late pregnancy and postpartum. During a short-term intervention based on high-protein, energy-restricted diet, Cotillard and colleagues observed an increase in gene richness, although only in individuals with low gene count at baseline [37]. Their findings suggested an antecorrelation between energy intake and microbial richness, opposite to the direction of effect observed in the present study, when considering changes from late pregnancy to postpartum. Similarly, results from a population-based Finish study showed a positive correlation between OTU richness and HbA1c levels [38], also opposite to the direction of effect observed in the present study. Although an effect of changes in diet and host metabolism cannot be ruled out, these results suggest the involvement of other factors in determining the change in microbial richness. One such factor could be the host immune system, which undergoes complex changes in the postpartum period [39].

So far, comparisons of intestinal microbiota during pregnancy and postpartum have not shown any differences in the abundance of taxa [8]. During a period of 3–16 months postpartum, it has been reported that 42 women with previous GDM had a relative lower abundance of *Firmicutes*, but this study did not have samples during pregnancy to compare with [40]. *Firmicutes* has also been seen in low abundance in cohorts of type 2 diabetes patients [6, 41].

Our findings suggest that gut microbiota of women with GDM have similarities with the microbiota reported in patients with type 2 diabetes and associated intermediary metabolic traits. For instance, the genus *Desulfovibrio* is enriched in patients with type 2 diabetes [6, 7]. Furthermore, the genus *Collinsella* has been associated with increased fasting levels of insulin and HOMA-IR in normoglycaemic pregnancies and is enriched in non-pregnant patients with type 2 diabetes [42, 43]. *Collinsella* has also been shown to decrease in abundance concurrent with a diet-induced weight loss in overweight individuals [44]. In infants, an increase in the abundance of *Collinsella* from birth to 6 months of age was associated with an increase in adiposity at 18 months of age [45].

*Collinsella* was also enriched in abundance of the gut microbiota postpartum in women with previous GDM, making the taxon a potential candidate in the search for bacteria that might contribute to later development of type 2 diabetes. Similarly, species of *Bacteriodes*, *Faecalibacterium* and *Isobaculum* were depleted in both third trimester and postpartum in women with GDM, and like *Collinsella*, these species could contribute to the long-term risk of type 2 diabetes in women with GDM.

At OTU level, reduced abundance of *Faecalibacterium* has previously been reported in the third trimester of healthy pregnant women [8]. In non-pregnant adults with metabolic syndrome and type 2 diabetes, *F. prausnitzii* is reported to be depleted as well [42, 46]. In the present study, we found *Faecalibacterium* OTUs both enriched and depleted in pregnant women with GDM. After adjustmen for pre-pregnancy BMI, we found an enrichment of one species assigned to *Faecalibacterium* in our GDM cohort. Furthermore, during pregnancy species of *Faecalibacterium* presented divergent associations with hsCRP, suggesting that different strains of *Faecalibacterium* are involved. An issue which can only be addressed by applying shot-gun sequencing-based metagenomics.

*Blautia* has been shown to be present with enriched abundance in glucose-intolerant individuals [47] and to be associated with metabolites reflecting an unhealthy metabolic state in individuals with a high BMI [38]. These findings are much in line with our results showing increased abundances associated with GDM, suggesting that enriched *Blautia* abundance goes together with a non-favourable metabolic profile. However, during pregnancy we found two species of *Blautia* associated with lower levels of plasma hsCRP, pointing to the occurrence of various subspecies of *Blautia* with opposite functionality related to host metabolism.

Similarly, *Ruminococcus2* abundance suggests a similarity between women with GDM and metabolic disorders in non-pregnant adults, as *Ruminococcus2* are enriched in type 2 diabetic patients [42], and *Ruminococcus gnavus* has been reported to be enriched in people with dysmetabolism and low microbial gene count [34]. Unexpectedly, we found that at the postpartum stage, the relative abundance of *Ruminococcus2* species decreased in the GDM group compared with the normoglycaemic group.

Investigating the relationships of metabolic traits and microbial taxa across groups led us to a surprising discovery of *Akkermansia* being associated with lower estimates of whole-body insulin sensitivity. *Akkermansia* has previously been reported to associate with improved metabolic health and to be inversely correlated to fasting plasma glucose and positively with insulin sensitivity [48–50]. In rodents, probiotics supplementation with *Akkermansia* improved glucose tolerance and insulin sensitivity [51]. Our results suggest that *Akkermansia* might have another impact on host physiology during pregnancy than otherwise described or that we find another subspecies of *Akkermansia*. The applied 16S rRNA gene amplicon sequencing methods does, however, not make it possible to investigate this finding at a deeper taxonomic resolution.

*Christensenella* is known to be highly heritable and to associate with low BMI [52]. Low abundance of *Christensenella* has been linked to a pre-diabetic health state and has also been associated with increased levels of acetate [38], a short chain fatty acid with an uncertain involvement in host metabolism and appetite regulation [53, 54]. In a rodent model, germ-free transplantation of *Christensenella* indicated a protective effect against weight gain [52]. We found that an OTU assigned to *Christensenella* was associated with higher fasting plasma glucose concentration, but the same *Christensenella* OTU was associated with lower weight gain during pregnancy. At the postpartum stage, a species of *Christensenella* was associated with lower levels of plasma hsCRP. All together, the findings suggest similarities between the features of *Christensenella* in pregnant and non-pregnant adults, but also different direction of association between abundance of *Christensenella* and host metabolic and inflammation phenotypes, indicating that the *Christensenella* species found in our study by 16S rRNA gene amplicon sequencing might not reflect the entire spectrum of taxa in the *Christensenellaceae* family.

When testing the association between different species and glucose metabolism, only a few OTUs were associated with different glycaemic traits. However, the four glycaemic traits used in our study represent different aspects of glucose metabolism [27], and physiologically there is no imperative requirement of overlap in features associated with different aspects of glucose metabolism. The fact that we only see a moderate overlap in OTUs associated with different glycaemic traits is not surprising, and might even be expected, given the different physiological features they represent.

Gut microbial communities are known to be affected by diet and weight in non-pregnant hosts [32, 37, 55]. The intestinal microbiota during pregnancy is associated with pre-pregnancy BMI as well as weight gain [30, 31]. When adjusting for pre-pregnancy BMI, we similarly demonstrated relationships between body composition and gut microbiota also during pregnancy. Our results confirm previous findings with enrichment of *Bacteroides* species in overweight pregnant women [31] and *Akkermansia* enriched in normal weight pregnant women [30, 31]. There was no overlap in taxonomic biomarkers of GDM with markers of overweight or obesity even though overweight is a risk factor for the development of GDM [29]. In our study, species assigned to *Christensenella* and *Alistipes* were associated with low weight gain and *Eisenbergiella* and *Lactobacillus* were associated with higher weight gain during pregnancy. This finding is concurrent with the findings in non-pregnant individuals as strains of *Eisenbergiella* and *Lactobacillus reuteri* have previously been associated with obesity [56, 57]. Of the taxa differentially abundant in women with and without GDM, only two were significantly correlated with pre-pregnancy BMI and only weakly so, indicating that the observed compositional differences were not strongly confounded by pre-pregnancy BMI.

To our knowledge, this is the first study investigating the gut microbiota composition at the time of GDM diagnosis. A few studies with few participants have examined gut microbiota either weeks before or after GDM diagnosis [8, 58]. Uniquely, for the present protocol, we could compare gut microbiota in the third trimester with postpartum samples allowing identification of taxa that exhibited differential abundance at the two time points according to maternal metabolic status during pregnancy. Another strength of the present protocol is that the gut microbiota of study participants was not confounded by antidiabetic drugs, a challenge recently demonstrated in microbiota studies of patients with type 2 diabetes [59]. A limitation of our study was that we did not have faecal samples before pregnancy. Also, our suggestion of subspecies occurrence with divergent functionalities calls for future shotgun-based sequencing studies of the intestinal microbiome in GDM.

## Conclusion

The intestinal microbiota composition of women with GDM differs from the microbiota of comparable normoglycaemic pregnant women in the third trimester. Intriguingly, the disrupted GDM gut microbiota has similarities with gut microbiota in individual patients with type 2 diabetes and associated intermediary metabolic dysfunctions. Outside pregnancy, at an average of 8.8 months after delivery, we still saw an aberrant intestinal microbiota of women with previous GDM. Prospective studies are warranted to explore whether such microbiota disruption conveys an increased risk of developing type 2 diabetes.

## Additional files

Additional file 1: **Figure S1.** Flow chart. **Figure S2.** Bristol stool scale and bowel movement frequency in women with and without GDM during pregnancy. **Figure S3.** Bristol stool scale and bowel movement frequency postpartum in women with and without previous GDM. **Figure S4.** Third trimester alpha diversity. **Figure S5.** Relationship between glycaemic traits and alpha diversity. **Figure S6.** Phylum level composition in pregnant women with gestational diabetes and with normal glucose regulation. **Figure S7.** Family-level composition in pregnant women with gestational diabetes and with normal glucose regulation. **Figure S8.** Genus-level composition in pregnant women with gestational diabetes and with normal glucose regulation. **Figure S9.** Bacterial operational taxonomic units associated with glycaemic traits during pregnancy. **Figure S10.** Bacterial operational taxonomic units associated with glycaemic traits during pregnancy adjusted for pre-pregnancy BMI. **Figure S11.** Frequency of pre-pregnancy overweight and obesity according to GDM status. **Figure S12.** Taxonomic biomarkers of overweight and obesity. **Figure S13.** Operational taxonomic units differentially abundant in pregnant women with normal and above normal pre-pregnancy body mass index. **Figure S14.** Operational taxonomic units differentially abundant in pregnant women with GDM and normal glucose regulation adjusted for pre-pregnancy BMI. **Figure S15.** Relationship between glycaemic traits and alpha diversity adjusted for pre-pregnancy BMI. (PDF 3075 kb)

Additional file 2: **Table S1.** Supplementary third trimester characteristics between pregnant women with GDM and normoglycaemic pregnant women. **Table S2.** Indications for OGTT. **Table S3.** Supplementary descriptive postpartum. **Table S4.** Nutrient intake during pregnancy and postpartum. **Table S5.** Operational taxonomic units differentially abundant in women with and without gestational diabetes during pregnancy and postpartum. **Table S6.** Operational taxonomic units associated with glycaemic traits in pregnant women independent of GDM status and unadjusted for pre-pregnancy BMI. **Table S7.** Higher order taxa associated with high sensitivity CRP. **Table S8.** OTUs associated with high-sensitivity C-reactive protein in laten pregnancy and postpartum. **Table S9.** Spearman correlations between GDM discriminant taxa and pre-pregnancy BMI. **Table S10.** Operational taxonomic units differentially abundant in overweigt (*n* = 67) and obese women (*n* = 58) compared with lean women (*n* = 61). **Table S11.** Operational taxonomic units differentially abundant in women with and without gestational diabetes with available pre-pregnancy BMI. **Table S12.** Operational taxonomic units associated with weight gain during pregnancy adjusted for pre-pregnancy BMI and gestational age. **Table S13.** Higher order taxa associated with gestational weight gain. **Table S14.** OTUs exhibiting differential change from the third trimester to postpartum dependent on GDM status. **Table S15.** Higher order taxa exhibiting differential change from the third trimester to postpartum dependent on GDM status. (XLSX 152 kb)

## Abbreviations

BMI: Body mass index
DNA: Deoxyribonucleic acid
GDM: Gestational diabetes mellitus
HOMA-IR: Homeostatic model of insulin resistance
hsCRP: High-sensitive C-reactive protein
IADPSG: International Association of the Diabetes and Pregnancy Study Group
LEfSe: Linear discriminant analysis effect size
OGTT: Oral glucose tolerance test
OTU: Operational taxonomic unit
rRNA: Ribosomal RNA gene
