# Supplementary material for: Gestational diabetes is associated with change in the gut microbiota composition in third trimester of pregnancy and postpartum
Source: Microbiome. 2018 May 15;6:89. doi: 10.1186/s40168-018-0472-x (PMC5952429; doi:10.1186/s40168-018-0472-x)
Supplement: Supplementary file 1 — Figure S1. Flow chart. Figure S2. Bristol stool scale and bowel movement frequency in women with and without GDM during pregnancy. Figure S3. Bristol stool scale and bowel movement frequency postpartum in women with and without previous GDM. Figure S4. Third trimester alpha diversity. Figure S5. Relationship between glycaemic traits and alpha diversity. Figure S6. Phylum level composition in pregnant women with gestational diabetes and with normal glucose regulation. Figure S7. Family-level composition in pregnant women with gestational diabetes and with normal glucose regulation. Figure S8. Genus-level composition in pregnant women with gestational diabetes and with normal glucose regulation. Figure S9. Bacterial operational taxonomic units associated with glycaemic traits during pregnancy. Figure S10. Bacterial operational taxonomic units associated with glycaemic traits during pregnancy adjusted for pre-pregnancy BMI. Figure S11. Frequency of pre-pregnancy overweight and obesity according to GDM status. Figure S12. Taxonomic biomarkers of overweight and obesity. Figure S13. Operational taxonomic units differentially abundant in pregnant women with normal and above normal pre-pregnancy body mass index. Figure S14. Operational taxonomic units differentially abundant in pregnant women with GDM and normal glucose regulation adjusted for pre-pregnancy BMI. Figure S15. Relationship between glycaemic traits and alpha diversity adjusted for pre-pregnancy BMI. (PDF 3075 kb) [file 40168_2018_472_MOESM1_ESM.pdf]

Supplementary figures

Figure S1; Flow chart

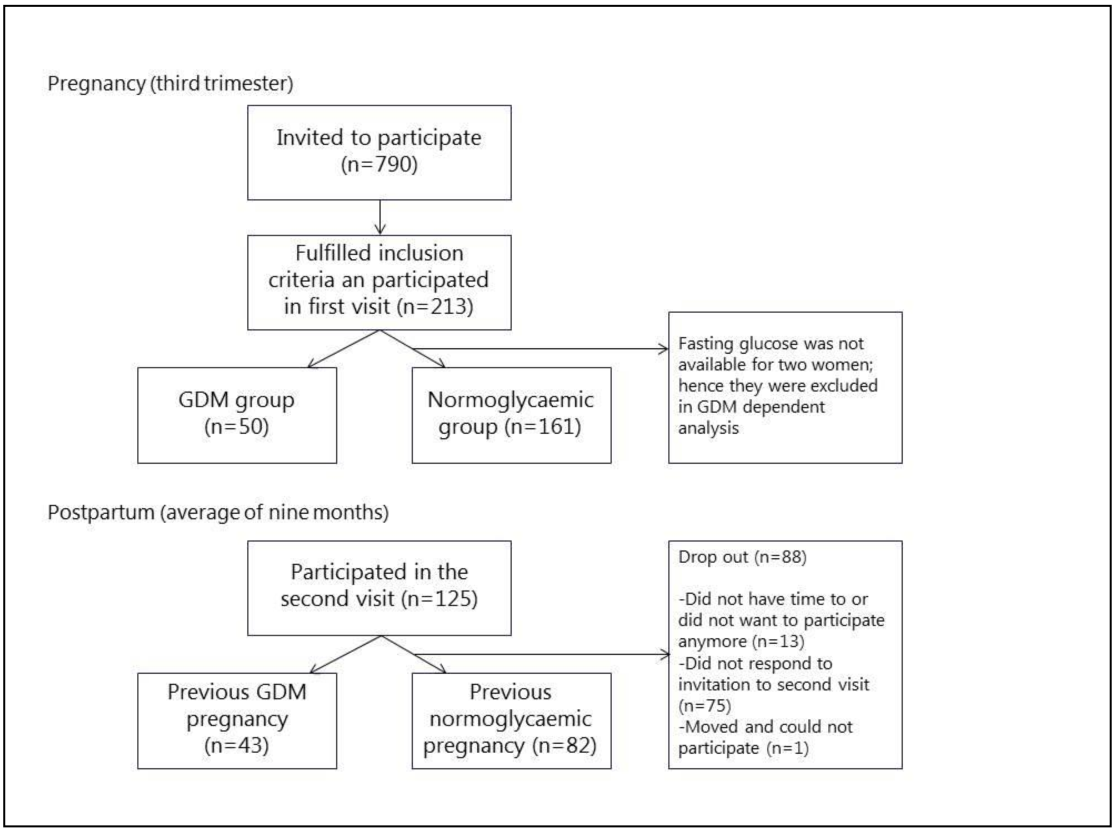

790 women were invited to participate in the project as they fulfilled the inclusion criteria. 213 women accepted and participated in the first project visit during third trimester. Two women was excluded as fasting plasma glucose was not available and the women was grouped according to the result of the oral glucose tolerant test (OGTT) in a glucose tolerant group (n=161) and a gestational diabetes mellitus (GDM) group (n=50). All participants were invited for a second project visit postpartum, 125 accepted, 82 women had had the previous normoglycaemic pregnancy and 43 women had had a previous GDM pregnancy. 86 women did not want to participate in the rest of the project due to personal reasons or had moved.

Figure S2; Bristol stool scale and bowel movement frequency in women with and without GDM during pregnancy

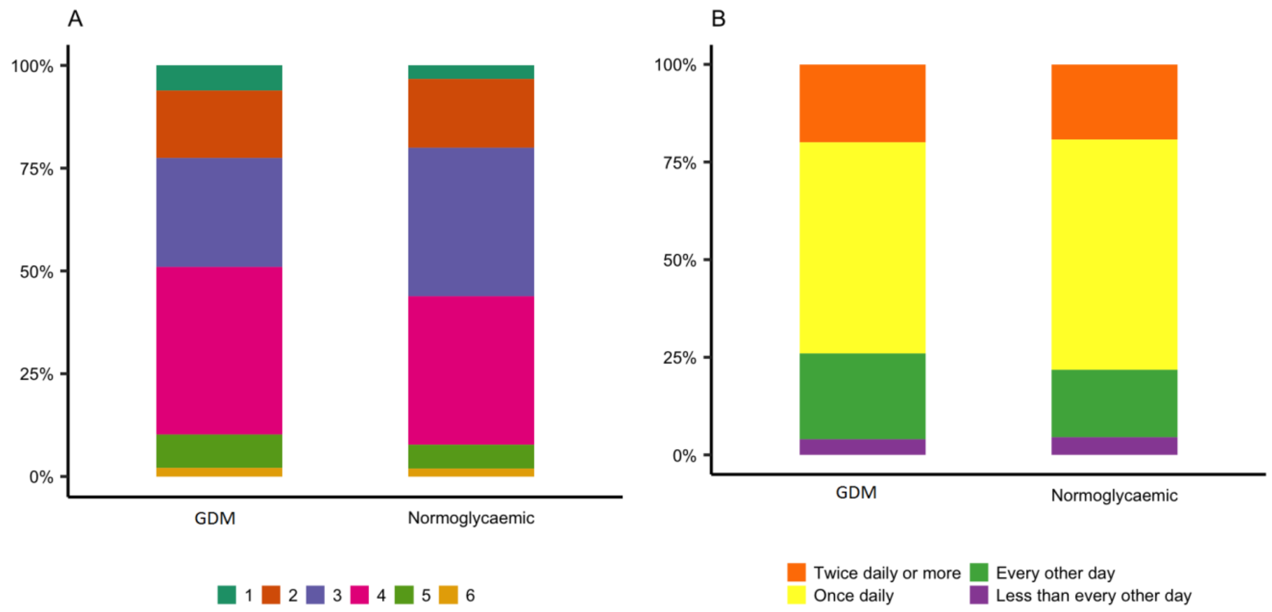

Figure S3; Bristol stool scale and bowel movement frequency postpartum in women with and without previous GDM

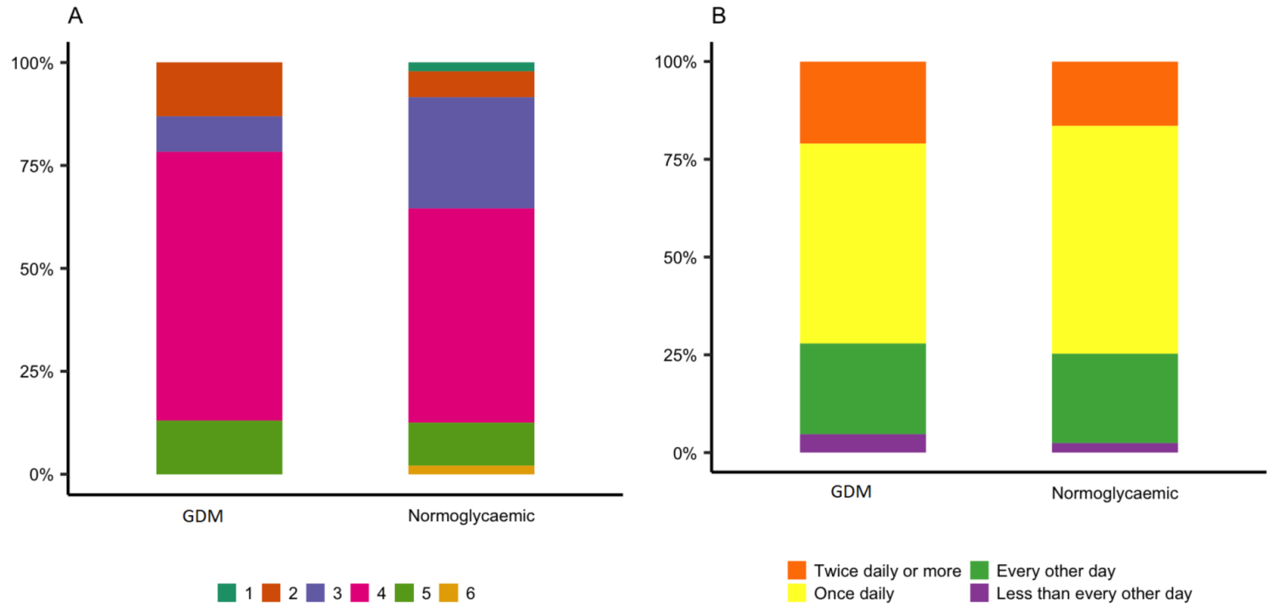

Figure S4. 3th trimester alpha diversity

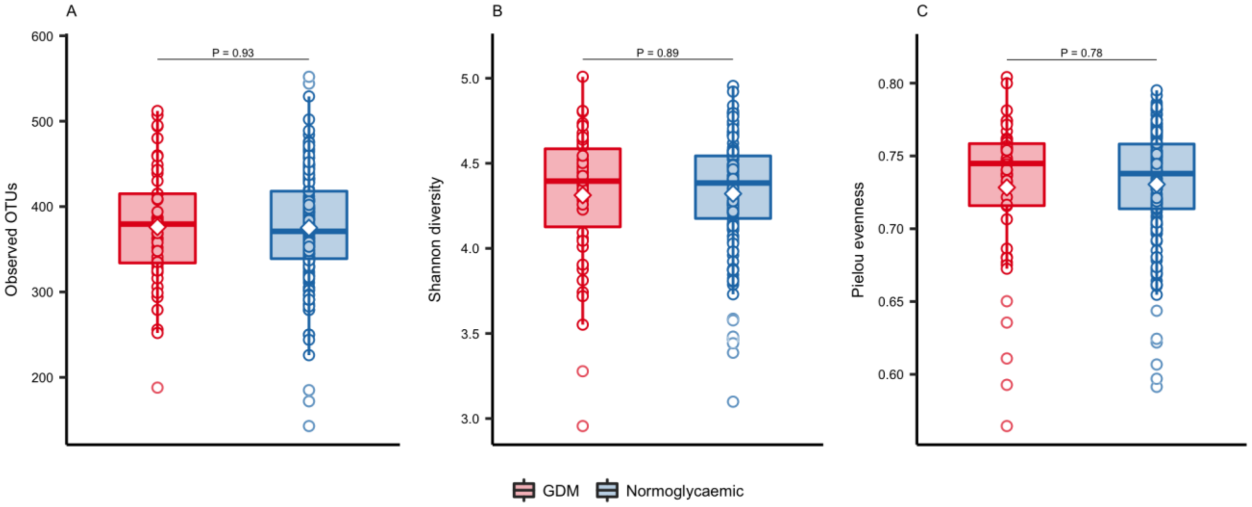

Alpha diversity in GDM and normoglycaemic pregnant women as represented by observed richness [A], Shannon diversity [B] and Pielou evenness [C] based on samples from GDM (diabetic n=50) and normoglycaemic (n=157) women during from 3rd trimester. Samples were rarefied to an equal sequencing depth of 10,000 reads. Boxes represent interquartile range (IQR), with the inside line representing the median. Whiskers represent values within 1.5×IQR of the first and third quartiles. Circles represent individual samples with lines connecting samples from the same individual. Differences between normoglycaemic and GDM pregnancies were tested using Students t-test. Difference in richness, Shannon diversity and Pielou evenness between time points in GDM and normoglycaemic women combined was tested using a paired t-test.

Figure S5. Relationship between glycaemic traits and alpha diversity

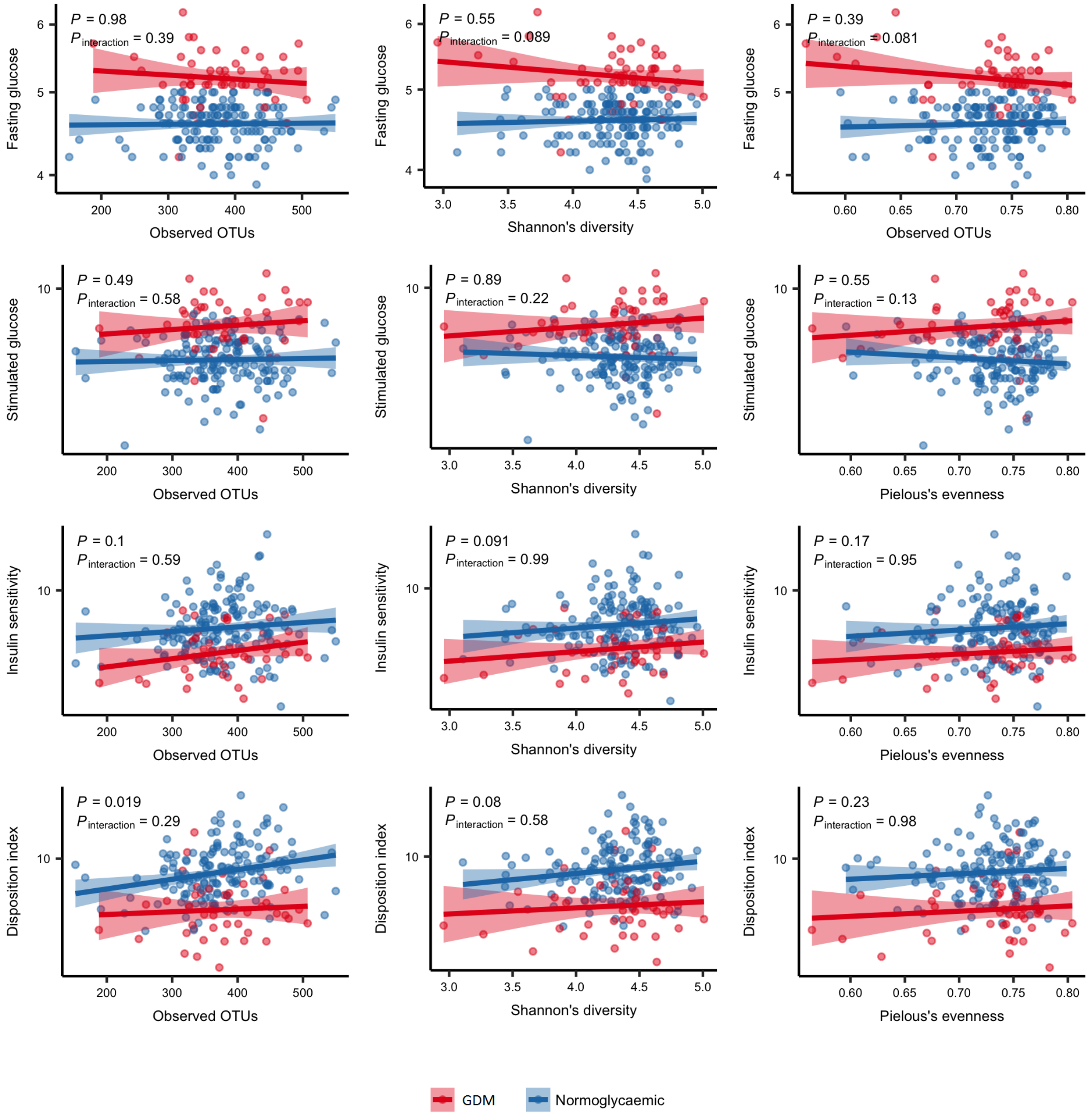

Scatter plots showing the relationships between four glycaemic traits (fasting and 2 h stimulated plasma glucose, insulin sensitivity, and disposition index; $\log_{10}$ scaled) and three measures of alpha diversity (observed OTUs, Shannon's diversity index, and Pielou's evenness index). Regression lines with 95% confidence intervals are plotted for women with GDM (here noted as red diabetic) and normoglycaemic women individualy. *P* indicate the nominal significance of the linear relationship between each glycaemic trait and alpha diversity measure (linear regression) in normoglycaemic women and women with GDM combined. $P_{interaction}$ indicate the nominal significance of the interaction between alpha diversity and GDM status for each combination of alpha diversity measure and glycaemic trait.

Figure S6. Phylum level composition in pregnant women with gestational diabetes and with normal glucose regulation

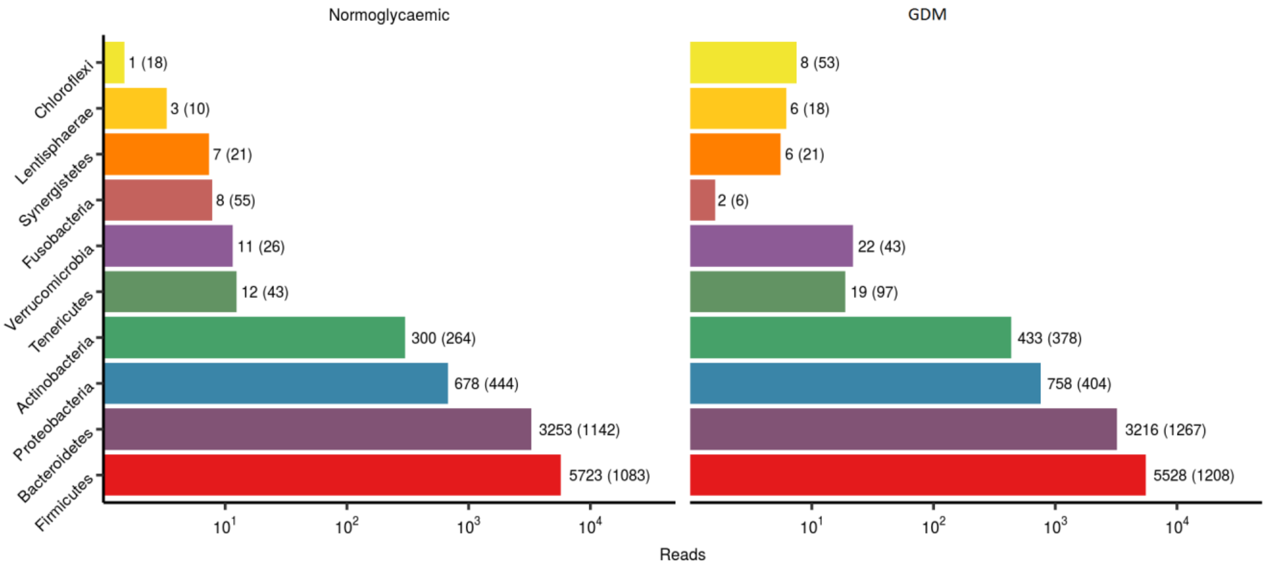

Mean (standard deviation) read count at a rarefied sequencing depth of 10,000 reads per samples. Only phyla with a mean read count ≥1 in women with gestational diabetes or in women with normal glucose regulation are depicted.

Figure S7. Family level composition in pregnant women with gestational diabetes and with normal glucose regulation.

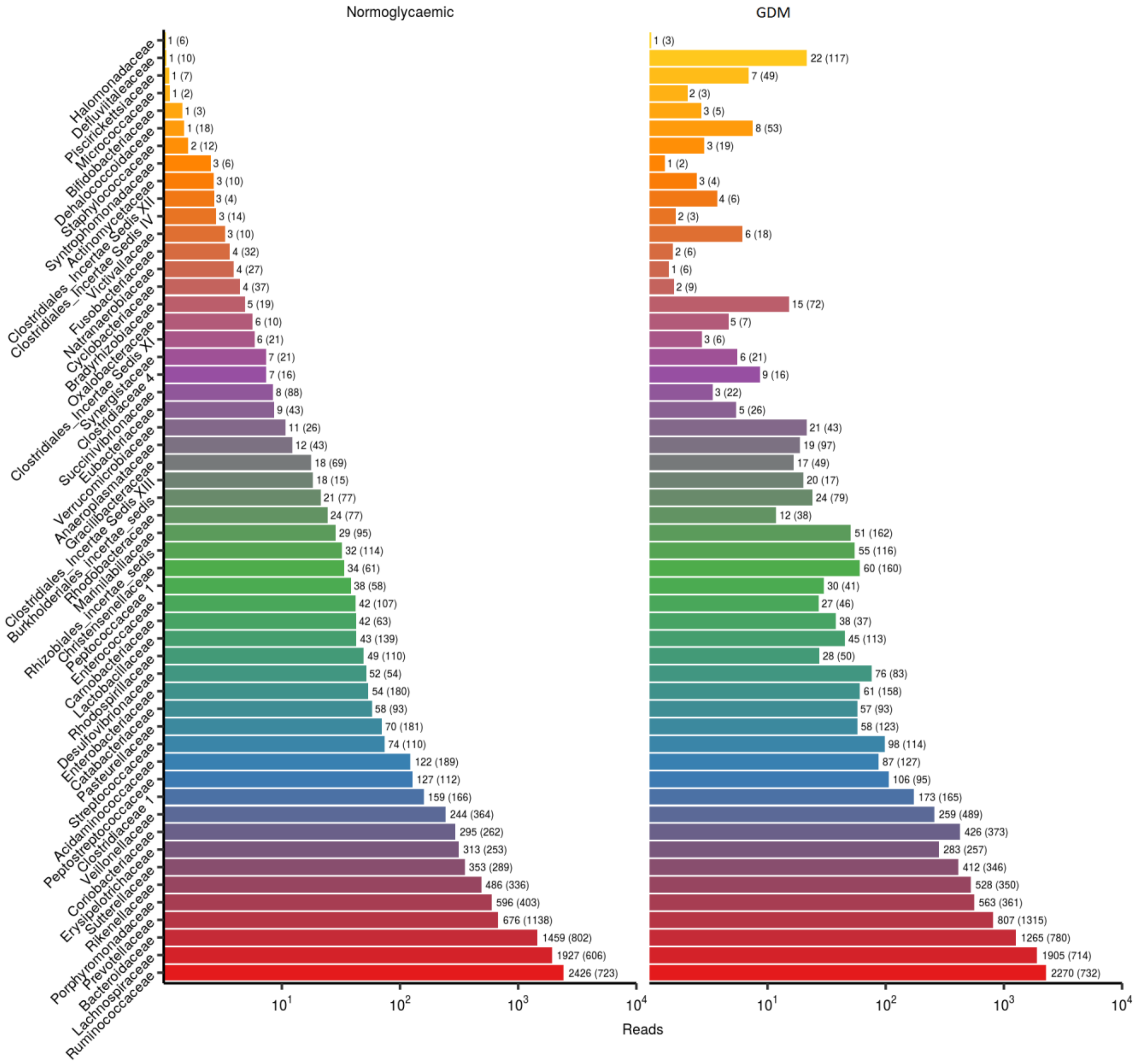

Mean (standard deviation) read count at a rarefied sequencing depth of 10,000 reads per samples. Only families with a mean read count ≥1 in women with gestational diabetes or in women with normal glucose regulation are depicted.

Figure S8. Genus level composition in pregnant women with gestational diabetes and with normal glucose regulation.

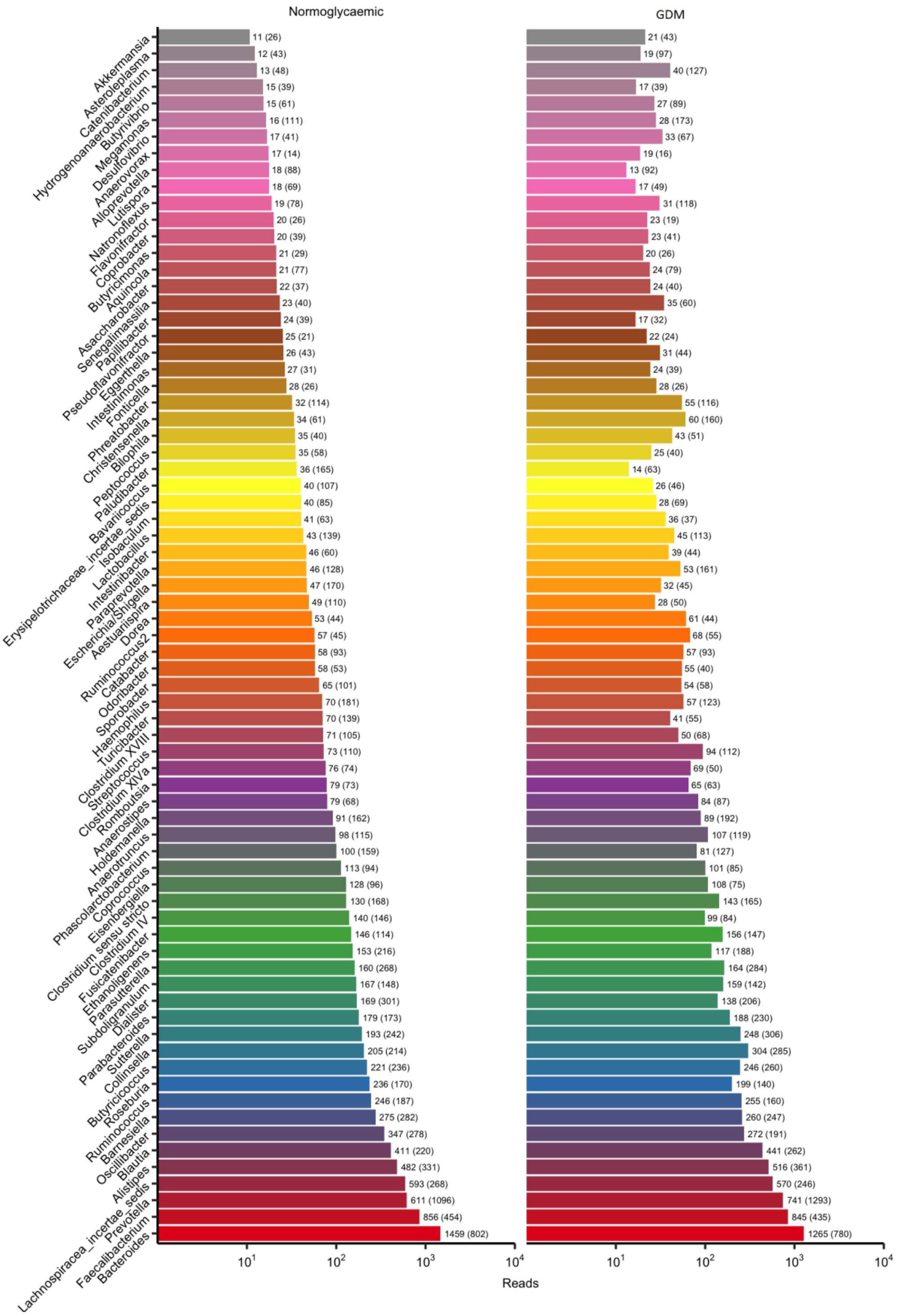

Mean (standard deviation) read count at a rarefied sequencing depth of 10,000 reads per samples. Only genera with a mean read count ≥10 in women with gestational diabetes or in women with normal glucose regulation are depicted.

Figure S9. Bacterial operational taxonomic units associated with glycaemic traits during pregnancy

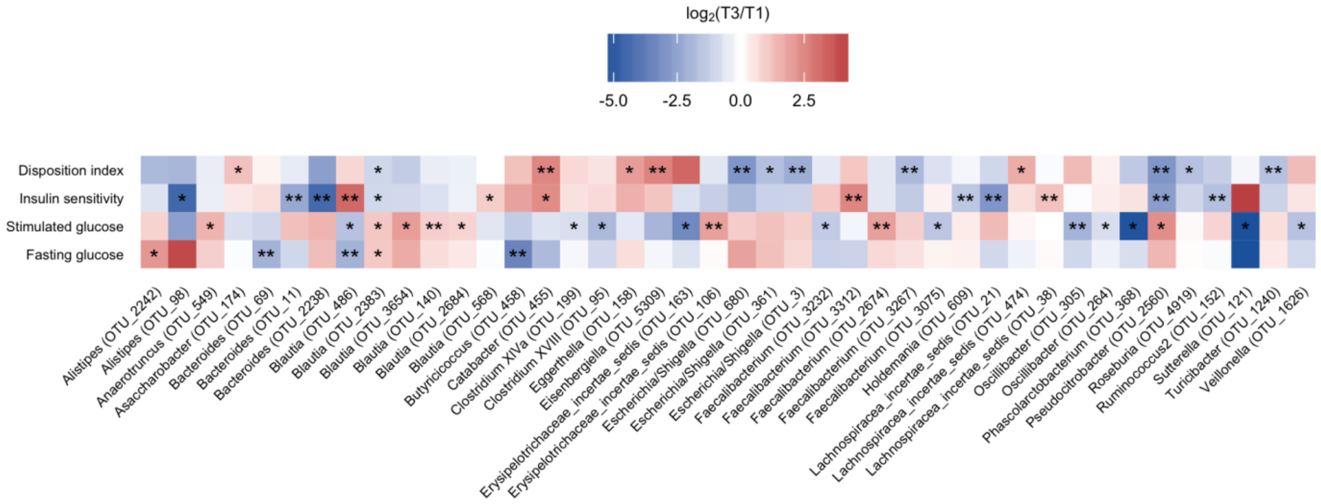

Samples were divided into tertiles based on fasting plasma glucose, stimulated 2h glucose, insulin sensitivity, and disposition index respectively. The plot depicts the $\log_2$ fold difference in operational taxonomic unit (OTU) abundance between women in the upper tertile (T3) compared to the lower tertile (T1) of each glycaemic trait tested using the negative binomial Wald test implemented in the *DESeq2* R package. Only OTUs significantly associated with either of the four traits at a 10% false discovery rate are depicted. Names are given at the genus level. OTUs are ordered alphabetically by genus annotation. Results in tabular form are available in Table S5. * Q<=0.1 ** Q<=0.05

Figure S10. Bacterial operational taxonomic units associated with glycaemic traits during pregnancy adjusted for pre-pregnancy BMI

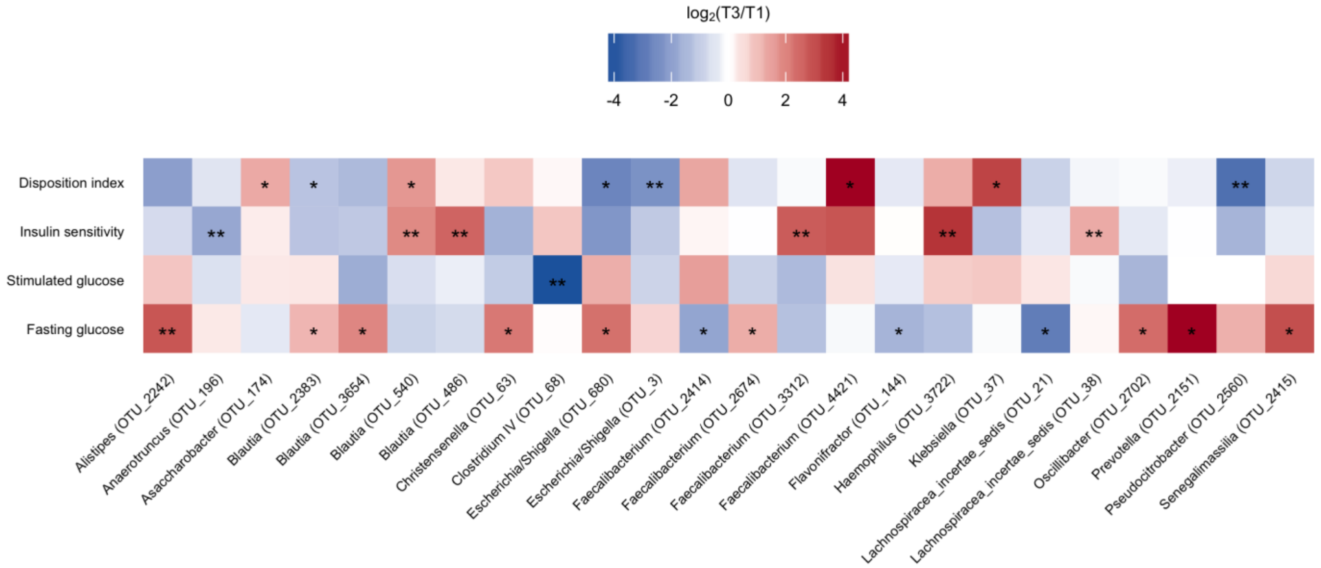

Samples were divided into tertiles based on fasting plasma glucose, stimulated 2h glucose, insulin sensitivity, and disposition index respectively. The plot depicts the log_2 fold difference in operational taxonomic unit (OTU) abundance between women in the upper tertile (T3) compared to the lower tertile (T1) of each glycaemic trait tested using the negative binomial Wald test implemented in the DESeq2 R package. Only OTUs significantly associated with either of the four traits at a 10% false discovery rate are depicted. Names are given at the genus level. OTUs are ordered alphabetically by genus annotation. Results in tabular form are available in Table S6. * Q<=0.1 ** Q<=0.05
.

Figure S11. Frequency of pre-pregnancy overweight and obesity according to GDM status

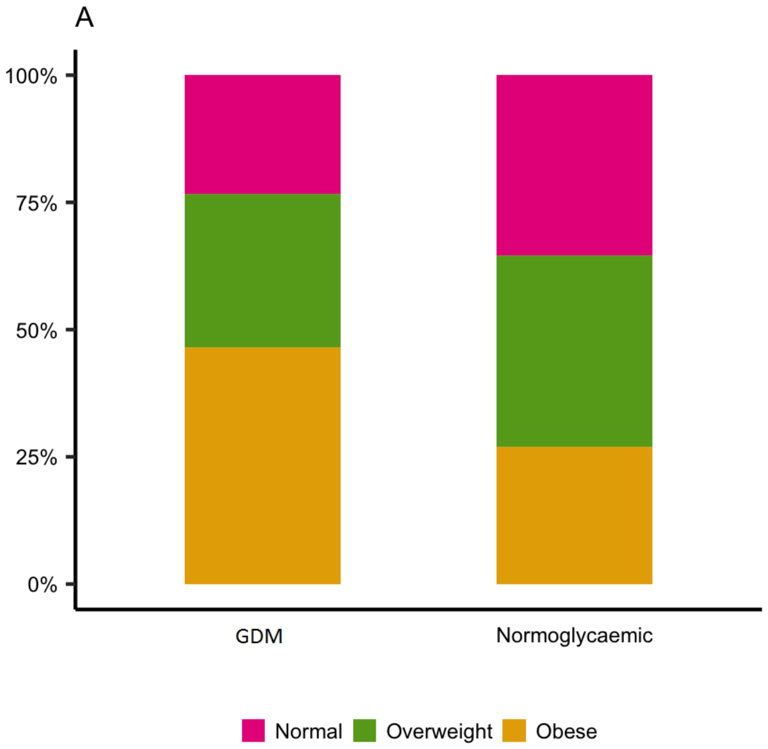

Figure S12. Taxonomic biomarkers of overweight and obesity

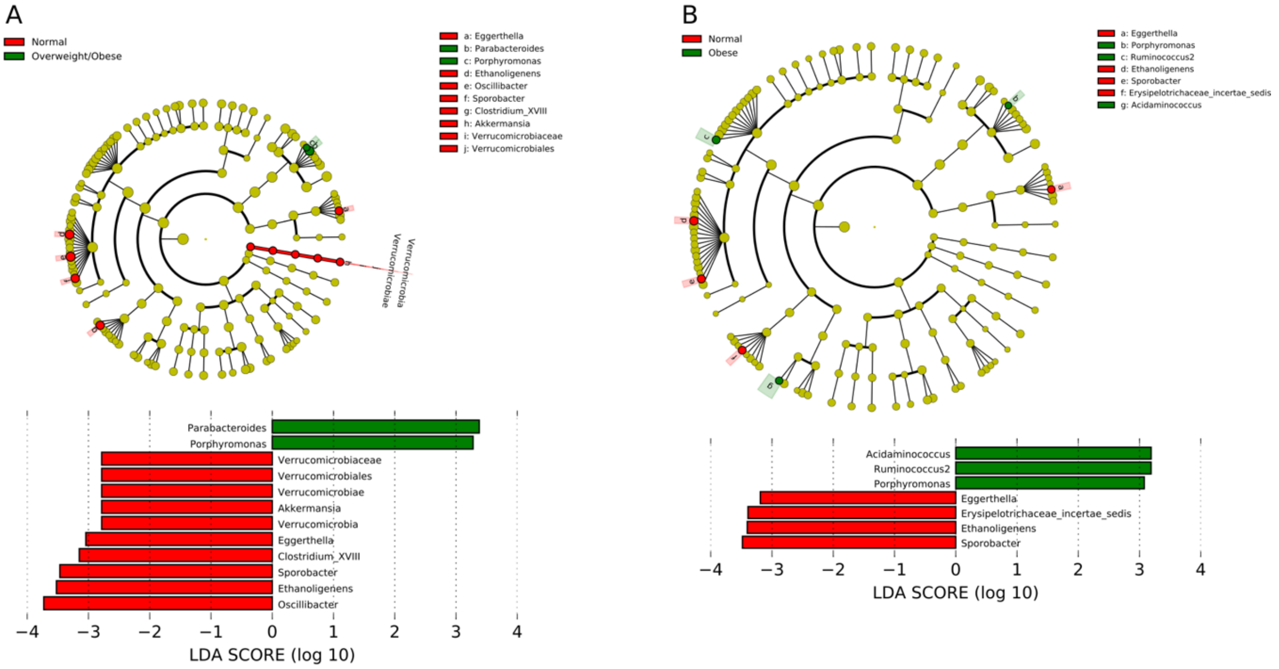

Cladogram and scores of taxonomic biomarkers of [A] above normal BMI (≥25 kg/m^2) and [B] obesity (≥30 kg/m^2) down to genus level identified by linear discriminant analysis (LDA) using LEfSe. Only women with information on pre-pregnancy body mass index was included in the analysis (n=185).

Figure S13. Operational taxonomic units differentially abundant in pregnant women with normal and above normal pre-pregnancy body mass index

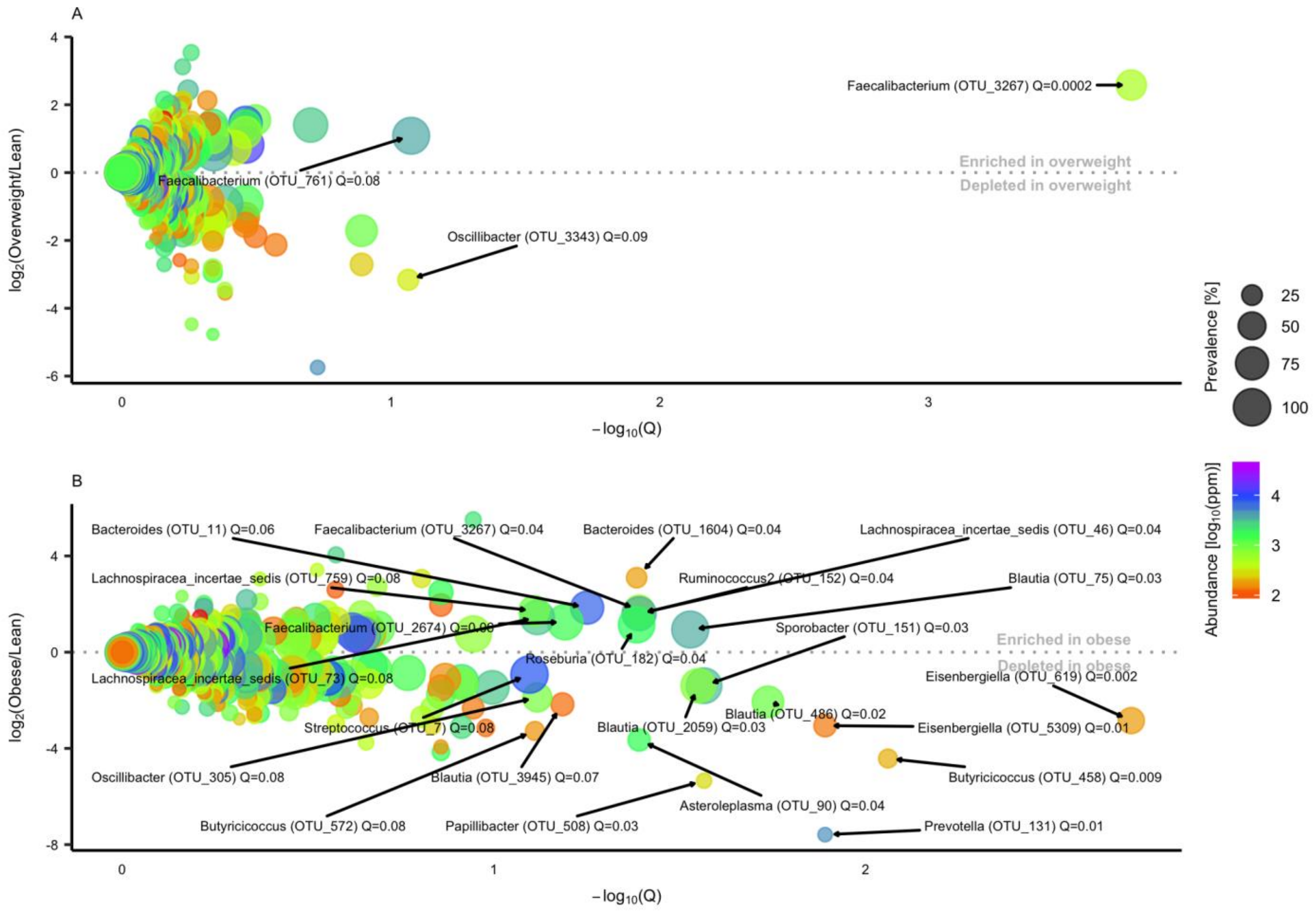

Volcano plot of estimated $\log_2$ fold difference in operational taxonomic unit (OTU) abundance between pregnant women with normal (<25) and [A] overweight (≥25) and [B] obese (>30) body mass index during third trimester of pregnancy. Only women with information about pre-pregnancy body mass index was included in the analysis (n=185).

Figure S14. Operational taxonomic units differentially abundant in pregnant women with GDM and normal glucose regulation adjusted for pre-pregnancy BMI

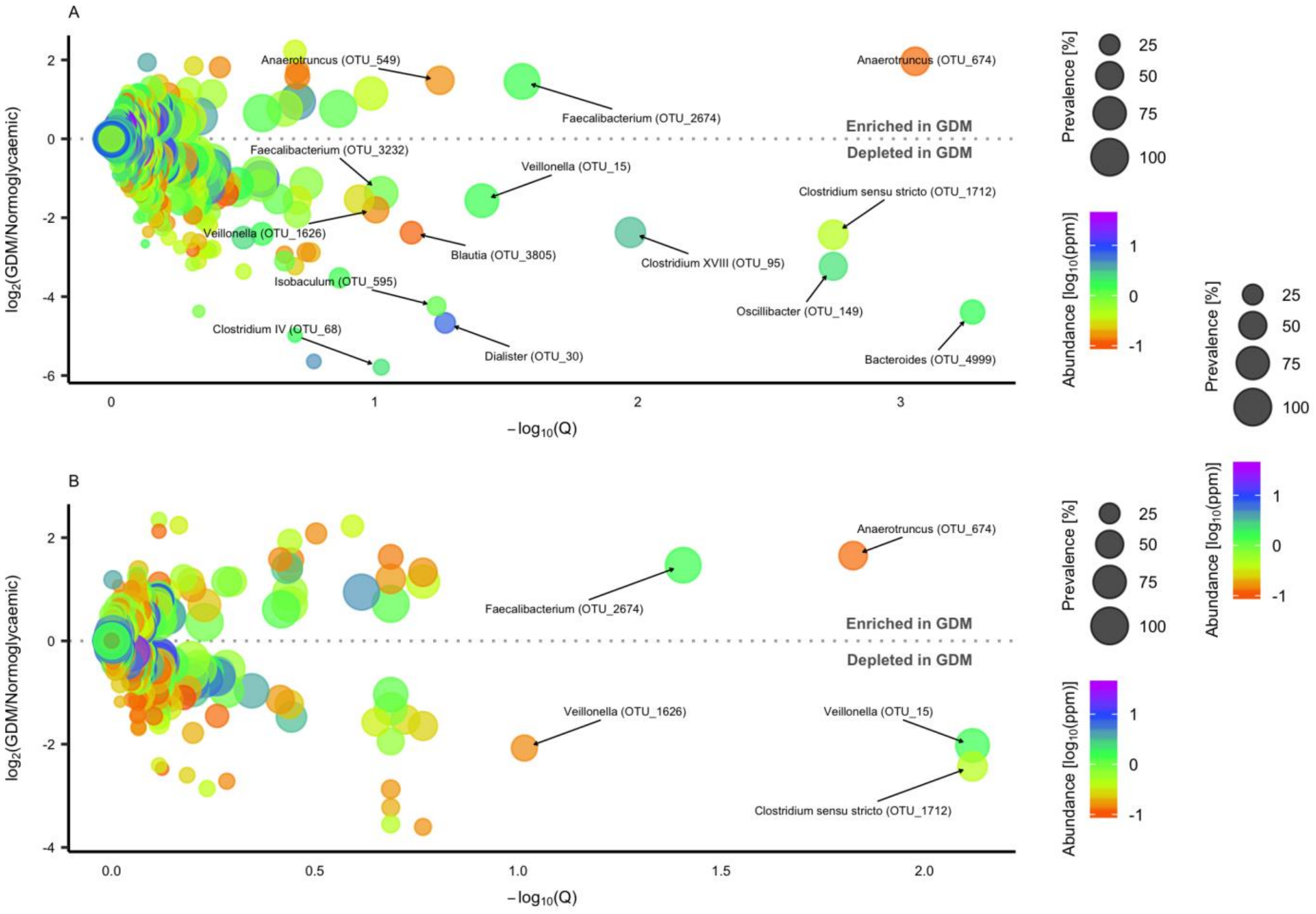

Volcano plot of estimated $\log_2$ fold difference in operational taxonomic unit (OTU) abundance between women with (n=43) and without gestational diabetes (n=143) during pregnancy from whom self-reported pre-pregnancy BMI was available [A] and adjusted for [B].

Figure S15. Relationship between glycaemic traits and alpha diversity adjusted for pre-pregnancy BMI

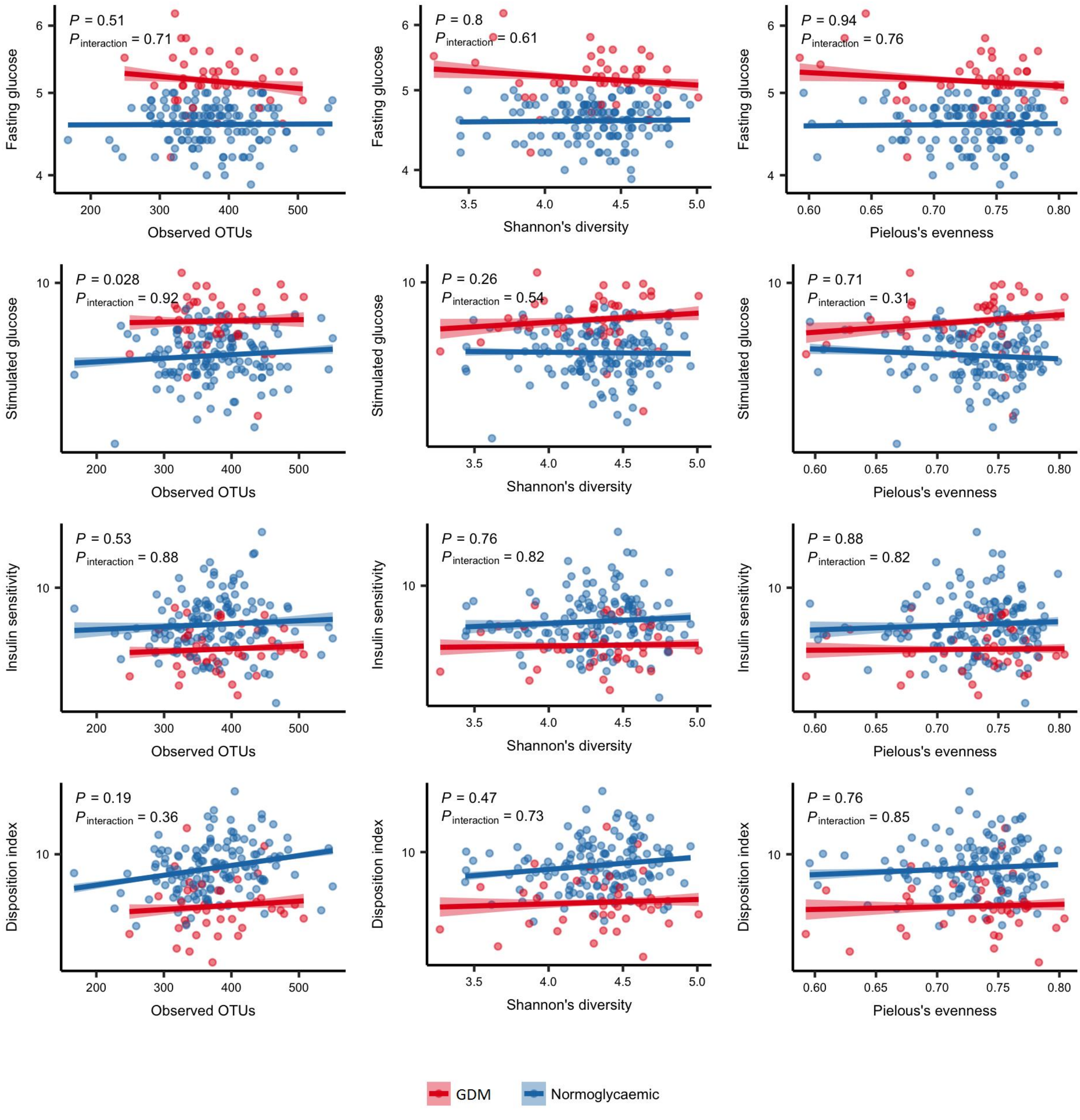

Scatter plots showing the relationships between four glycaemic traits (fasting and 2 h stimulated plasma glucose, insulin sensitivity, and disposition index; $\log_{10}$ scaled) and three measures of alpha diversity (observed OTUs, Shannon's diversity index, and Pielou's evenness index). Regression lines adjusted for pre-pregnancy BMI with 95% confidence intervals are plotted for women with GDM (here in red noted as diabetic) and normoglycaemic women individually. *P* indicate the nominal significance of the linear relationship between each glycaemic trait and alpha diversity measure adjusted for pre-pregnancy BMI (linear regression) in normoglycaemic women and women with GDM combined. $P_{interaction}$ indicate the nominal significance of the interaction between alpha diversity and GDM status adjusted for pre-pregnancy BMI for each combination of alpha diversity measure and glycaemic trait.
